# A novel transgenic mouse line with hippocampus-dominant and inducible expression of truncated human tau

**DOI:** 10.1186/s40035-023-00379-5

**Published:** 2023-11-10

**Authors:** Yang Gao, Yuying Wang, Huiyang Lei, Zhendong Xu, Shihong Li, Haitao Yu, Jiazhao Xie, Zhentao Zhang, Gongping Liu, Yao Zhang, Jie Zheng, Jian-Zhi Wang

**Affiliations:** 1 Department of Pathophysiology, Key Laboratory of Ministry of Education for Neurological Disorders, School of Basic Medicine, Tongji Medical College, Huazhong University of Science and Technology, Wuhan, 430030 China; 2 Department of Neurology, Renmin Hospital of Wuhan University, Wuhan, 430030 China; 3 Key Laboratory of Ministry of Education for Neurological Disorders, Department of Endocrine, Liyuan Hospital, Tongji Medical College, Huazhong University of Science and Technology, Wuhan, 430077 China; 4 Neuroscience Research Institute and Department of Neurobiology, School of Basic Medical Sciences, Peking University, Beijing, China; 5 Key Laboratory for Neuroscience, Ministry of Education/National Health Commission, Peking University, Beijing, 100083 China; 6 Co-Innovation Center of Neuroregeneration, Nantong University, Nantong, 226000 China

**Keywords:** Alzheimer’s disease, Animal model, Tau hyperphosphorylation, Truncated tau, Tet-on system

## Abstract

**Background:**

Intraneuronal accumulation of hyperphosphorylated tau is a defining hallmark of Alzheimer’s disease (AD). However, mouse models imitating AD-exclusive neuronal tau pathologies are lacking.

**Methods:**

We generated a new tet-on transgenic mouse model expressing truncated human tau N1-368 (termed hTau368), a tau fragment increased in the brains of AD patients and aged mouse brains. Doxycycline (dox) was administered in drinking water to induce hTau368 expression. Immunostaining and Western blotting were performed to measure the tau level. RNA sequencing was performed to evaluate gene expression, and several behavioral tests were conducted to evaluate mouse cognitive functions, emotion and locomotion.

**Results:**

Dox treatment for 1–2 months at a young age induced overt and reversible human tau accumulation in the brains of hTau368 transgenic mice, predominantly in the hippocampus. Meanwhile, the transgenic mice exhibited AD-like high level of tau phosphorylation, glial activation, loss of mature neurons, impaired hippocampal neurogenesis, synaptic degeneration and cognitive deficits.

**Conclusions:**

This study developed a well-characterized and easy-to-use tool for the investigations and drug development for AD and other tauopathies.

**Supplementary Information:**

The online version contains supplementary material available at 10.1186/s40035-023-00379-5.

## Introduction

Alzheimer^’^s disease (AD) is the most common cause of dementia in the elderly, which affects over 50 million people worldwide [1, 2]. Intraneuronal formation of neurofibrillary tangles (NFTs) composed of hyperphosphorylated tau protein is a pathological hallmark of AD [3, 4] and is well recognized to correlate with cognitive deficits of patients [4, 5]. Therefore, increasing attention has been paid on the mechanisms underlying how tau pathology contributes to AD, as well as the tau-targeted drug discovery [6–9]. However, studies of tau pathology and drug development are largely limited by the lack of readily available mouse models that mimic the AD-specific tau pathology.

Several tau transgenic (Tg) mouse models have been used for decades in the research of tau pathology [10], including: (1) lines overexpressing wild-type (WT) or mutant human tau (hTau), such as ALZ17 (WT) [11] and PS19 (P301S) [12]; (2) Tg mice with expression of full-length WT or mutant hTau in the absence of endogenous murine tau, such as the hTau line (JAX005491) [13–15]; and (3) mice with expression of WT or mutant full-length hTau under the induction of tetracycline, such as rTg4510 (CaMK2a:P301L) [16] and rT2 (Col1a1:P301L) [17]. However, these Tg lines show limitations in imitating the tau pathology in AD [18]. First, most non-inducible hTau-Tg lines do not exhibit overt phosphorylated tau accumulation in the brain until 6–9 months of age [11, 19], so researchers have to spend much time and resources to keep mice before experiments. Second, the commonly studied tau mutations in tau lines such as P301L and P301S are only found in frontotemporal dementia but not in AD patients [20, 21]. In addition, some other mutated tau lines like rTg4510 (CaMK2a:P301L) show serious motor impairments, which may disturb the evaluation of cognitive functions in behavioral tests [17, 22–24].

To generate an easy-to-use animal model with accurate simulation of the actual AD tau pathology, we generated a novel hTau368-Tg mouse line, in which the AD-like truncated hTau N1–368 (hTau368) was expressed under the neuron-specific enolase 2 (Eno2) promoter with induction of tetracycline (tet-on). The neurotoxic tau fragment N1–368 is cleaved by asparagine endopeptidase (AEP), and it accumulates and mediates NFT formation during aging and AD [25]. Recently, we have used hTau368 mice as a model to test the effectiveness of a peptide drug designed to specifically facilitate tau dephosphorylation [26]. For better use of this model, we here report more detailed characterization of AD-like pathologies in this mouse line.

## Materials and methods

### Animals

All mice were housed in groups of three to four per cage, under a 12 h light/dark cycle at 23–25 °C. Food and water were available ad libitum. Doxycycline hyclate (Beyotime, ST0398) was dissolved in drinking water (2 mg/l) and administered ad libitum. Equal numbers of male and female mice were randomly assigned to vehicle (Veh) or dox-treatment (Dox) groups. Mice were sacrificed at age of 7 months unless otherwise specified. All experiments and data analyses were conducted by experimenters blind to the groupings. All animal experiments were conducted in accordance with relevant ethical regulations for animal testing and research, and were approved by institutional guidelines and the Animal Care and Use Committee of Tongji Medical College, Huazhong University of Science and Technology.

### Tg hTau368 generation

The hTau368 mice were generated jointly by our laboratory and Nanjing Biomedical Research Institute of Nanjing University. Specifically, an Eno2-h*MAPT* vector, Insulator-pTRE3G-Kozak-ATG-hMapt N1-368-CDS-TGA-polyA-bGH-polyA-TAA-rtTA3G-Kozak-ATG-Rat Eno2 promoter-Insulator, was constructed by linking reactive cloning. pTRE is a tetracycline-induced promoter. h*MAPT* is the targeted gene searched through https://www.ncbi.nlm.nih.gov/gene query (Gene ID: 4137). *MAPT* contains 12 transcripts, of which the 2N4R transcript NM_001123066.4 and the sequences encoding tau N1-368 were selected. PolyA was used as the termination signal. rtTA3G was the tetracycline-regulated transcription activator. Rat Eno2 promoter (Gene ID: 24,334, transcript xM_0062373304) was used to control the expression of rtTA3G.

The Eno2-h*MAPT* vector was confirmed by DNA sequencing and microinjected into the nucleus of fertilized eggs of C57BL/6 J mice. Surviving fertilized eggs were implanted into the uterus of pseudo-pregnant C57BL/6 J mice. Founder mice in the F0 offspring were identified by PCR at 1 month old. Whole-genome sequencing was performed to find the insertion site of the targeted gene (Additional file 1: Fig. S1). Identification of homozygous and hemizygous hTau368 was conducted by PCR using primers shown in Additional file 1: Table S1. F1 hTau368 mice were used for expand reproduction and F2–4 offspring with transgene were used for experiments in this study. C57BL/6 J mice were used as wild-type controls.

### Antibodies

Antibodies used in the present study are summarized in Additional file 1: Table S2. The polyclonal rabbit anti-TauN368 antibody gifted by Professor Keqiang Ye was developed in his laboratory and had been reported previously [25]. The new monoclonal mouse anti-tauN368 antibody was jointly developed by AtaGenix (Wuhan, China). In brief, a keyhole limpet hemocyanin (KLH)-conjugated tau peptide Cry-_358_DNITHVPGGGN_368_ was synthesized and used as an antigen to immunize C57BL/6 J mice for 4 times within two months. Antisera were pooled and the immunoreactivities to tauN368 and full-length hTau were tested by ELISA. Two mice with well serum immunization validated by Western blotting were selected, whose spleens were harvested for cell fusion. Positive hybridoma cells were selected by hypoxanthine-aminopterin-thymidin (HAT) medium, and the positive hybridoma cells were subcloned by limited dilution method to obtain monoclonal cell lines. During each subcloning, indirect ELISA screening was performed for 2–3 rounds to obtain positive monoclonal cell lines and then the antibody subtypes of the constructed cell lines were identified. Balb/c mice were injected with selected cell line, and ascitic fluid was purified for TauN368 antibody (titer > 1:64,000). All TauN368 Western blotting results were obtained using the newly generated antibody, while all TauN368 immunostaining results were obtained using the polyclonal rabbit anti-TauN368 antibody.

### Western blotting

Mouse brains were removed, and the cortex and hippocampus were dissected on ice, respectively. Samples were homogenized with RIPA lysis buffer containing 50 mM Tris (pH 7.4), 150 mM NaCl, 1% Triton X-100, 1% sodium deoxycholate, and 0.1% SDS (#P0013B, Beyotime, Shanghai, China) mixed with a cocktail of protease and phosphatase inhibitors (Thermo Scientific, Waltham, MA), at a ratio of 10 μl/mg tissue. Then the tissue homogenates were centrifuged for 20 min at 12,000 ×*g*, resulting in RIPA-soluble and RIPA-insoluble parts. Protein concentration in the RIPA-soluble lysate was quantitated using BCA protein assay kit (Thermo Fisher), and equal amounts of proteins were loaded on SDS–PAGE gels. The RIPA-insoluble pellets were further dissolved in 90–120 μl of loading buffer containing 200 mM Tris–HCl pH 6.8, 8% SDS and 40% glycerol. Proteins were separated by SDS-PAGE (10%), transferred onto nitrocellulose membranes (Merck Millipore, Darmstadt, Germany) and then blocked with 5% bovine serum albumin (BSA). Membranes were incubated with primary and secondary antibodies (Additional file 1: Table S2), in sequence. Blots were visualized by an enhanced chemiluminescence substrate system (Santa Cruz Biotech, Santa Cruz, CA), imaged by an Odyssey Imaging System (LI-COR Biosciences, Lincoln, NE), and quantified by the ImageJ software. β-Actin was used as a loading control.

### Immunostaining and quantification

Mice were anesthetized with 2% isoflurane (RWD Life Science, Shenzhen, China) one day after completion of the final behavioral trial, and perfused via the ventriculus sinister with 0.9% NaCl for 5 min followed by PBS containing 4% paraformaldehyde for 5 min. Brains were cryoprotected sequentially in 25% and 30% sucrose, for 2 days in total, and then cut into 30-μm sections using a cryostat microtome (CM1900, Leica, Wetzlar, Germany). For immunohistochemistry, free floating sections were immersed in 3% H_2_O_2_ in anhydrous methanol for 30 min, and non-specific sites were blocked with BSA for 30 min at room temperature. Brain slices were then incubated overnight at 4 °C with primary antibodies. Immunoreactions were developed using a DAB-staining kit (ZSGB-BIO, Beijing, China). Images were taken by an automatic slice scanning system (SV120, Olympus, Tokyo, Japan) at 20 × magnification and analyzed with ImageJ software. Areas of various regions of the brain were measured.

For immunofluorescence, sections were thoroughly washed with PBST (PBS containing 0.1% Triton X-100) and incubated overnight with primary antibodies under 4 °C. After that, the sections underwent PBST washes for 15 min, followed by 1-h incubation with the secondary antibody at 37 °C, and finally counterstained with DAPI. Images were taken by an automatic slice scanning system (SV120, Olympus) and a two-photon laser-scanning confocal microscope (LSM 800, Zeiss, Oberkochen, Germany) at 20 × magnification, and analyzed with the ImageJ software. The areas of hTau368 staining in various regions, the mean intensity of NeuN staining in the CA1 pyramidal layer, as well as the numbers of cells positive for MAP2, GFAP, Iba1 or DCX staining were measured or counted in a single 20 × magnification view and averaged per 0.1 mm^2^. One section from each mouse and a total of four to six mice per group were analyzed.

### Gallyas silver staining

Free-floating brain sections were washed with Tris-buffered saline (TBS) for 3 × 5 min, and then placed in 5% periodic acid followed by alkaline silver iodide solution and developer solution (#G1052, Servicebio, Wuhan, China). After washing with acetic acid and water, they were placed in 0.1% gold chloride, followed by sodium thiosulphate solution. The sections were placed in absolute ethanol for 3 × 5 min and in xylene for 2 × 5 min to become transparent. Images were taken by an automatic slice scanning system (SV120, Olympus, Tokyo, Japan) and analyzed at 100 × magnification.

### Thioflavin S staining

Free-floating brain sections were washed with TBS for 3 × 5 min, and then incubated with 0.3% Thioflavin S (#1326-12-1, Sigma, Darmstadt, Germany) dissolved in 50% ethanol. The sections were then decolorized in 50% ethanol for 3 × 5 min, washed in TBS, and subsequently co-stained with DAPI for 10 min.

### Sholl analysis

Fluorescent images of GFAP and Iba1 immunostaining were analyzed as reported previously [27]. Processes were traced by the plugin Sholl Analysis in ImageJ. The intersections of processes were counted within concentric circles at 10-μm intervals from the center of the soma. Ending radius and the sum of intersections were used to indicate the complexity of processes.

### Electron microscopy

Synaptic density and neuronal axon width were determined by electron microscopy as described previously [28]. After deep anesthesia, mice were perfused transcardially with 2% glutaraldehyde and 3% paraformaldehyde in PBS. Hippocampal slices were post-fixed in cold 1% OsO_4_ for 1 h. Samples were prepared and examined using standard procedures. Ultrathin sections (90 nm) were stained with uranylacetate and lead acetate and viewed at 200 kV in a Tecnai electron microscope. The slices were from three mice in each group. Synaptic contacts were identified by the presence of synaptic vesicles and post-synaptic densities with high electron density.

### Transcriptomic analysis

Hippocampal tissues were isolated on ice. The detailed procedures of total RNA extraction, mRNA library construction and quality assurance has been reported previously [29]. The differentially expressed genes (DEG) between vehicle-treated (*n* = 3) and Dox-treated hTau368 mice (*n* = 3) (FDR-adjusted* P* < 0.05, fold change > 1.5) were analyzed by DESeq2 (v1.4.5) [30]. Kyoto encyclopedia of genes and genomes (KEGG) enrichment and the relationship network of KEGG pathways of DEGs were analyzed by Dr. Tom software (BGI, Shenzhen, China). Gene network analysis was performed using the STRING database (https://string-db.org/).

### Novel location recognition test

Mice were handled and habituated before tests. On day 1, each mouse was placed in the center of a plastic box in which two identical objects (A and B) were located in two corners. The mouse was allowed 5 min to explore freely. After 24 h, the mouse was placed back to the box with object A in the same corner while object B placed in a new location. Again, each mouse was allowed 5 min to explore. The time the mouse spent exploring objects A and B was recorded as T_A_ and T_B_, respectively. Exploration was identified by a video tracking system (Anymaze Technology SA, Stoelting Co., IL) once the mouse head stayed close (< 3 cm) to either object. Mice with T_A_ or T_B_ less than 2 s were excluded from analysis. The discrimination index was calculated as (T_B−_T_A_)/(T_B_ + T_A_). A higher discrimination index indicates better spatial memory.

### Morris water maze test

Mice were kept in the test room for 24 h before tests. In the learning phase, mice were trained in the maze to find the hidden platform. The learning phase consisted of 3 trials per day with an interval of 30 min, from 14:00 to 17:00, for 5 consecutive days. In each trial, a mouse was placed in one of the three quadrants without the platform, facing the wall of the pool. If the mouse found the hidden platform within 60 s, another 15 s was left for learning consolidation. If the mouse did not find the platform within 60 s, it was guided to the platform and allowed to stay on the platform for 15 s. The time to find the platform during the 5-day training was recorded as the escape latency. On day 6, a testing trial was performed. The hidden platform was removed and each mouse was placed in the quadrant opposite to the target quadrant. The time/distance and trajectory of each mouse traveling in the pool were recorded and analyzed by a video tracking system (Chengdu Taimeng Software Co., Ltd, China). Mice disabled in eyes or limbs were excluded from analysis.

### Open field test

Mice were handled for 1 day, and placed in the test room the day before the behavioral test to get acclimated to the environment. The open field apparatus was a 60 × 60 × 50 cm^3^ white plastic box, with the floor divided virtually into 16 equal squares in the monitoring system, including a central field (the central 4 square regions) and 12 periphery fields. Each mouse was allowed to explore freely in the box for 5 min. The time and distance each mouse travelled in different zones were recorded and analyzed by the ANY-maze video tracking system (Stoelting Co., WoodDale, IL).

### Elevated-plus maze test

The elevated-plus maze consisted of two enclosed arms (65 × 5 × 20 cm^3^) and two open arms (65 × 5 cm^2^). The apparatus was elevated to 50 cm above the floor. Each mouse was placed in the center of the maze, facing the open arm opposite to the experimenter, and allowed to explore freely for 5 min. The time and place mice traveled in the maze were recorded and analyzed using a video tracking system (Chengdu Taimeng Software Co., Ltd, China).

### Statistical analyses

All data were analyzed and plotted using GraphPad Prism 8 (La Jolla, CA). Comparisons between two groups were made by two-tailed unpaired Student’s *t*-tests, and comparisons between multiple groups were conducted with one-way, two-way or repeated measures ANOVA followed by *post-hoc* tests for multiple comparisons. *P* < 0.05 was considered statistically significant. All experiments and analysis were performed in a blind manner. All values are shown as mean ± SEM or min-median-max unless otherwise specified.

## Results

### Generation of the hTau368 transgenic mice

**Fig. 1:**
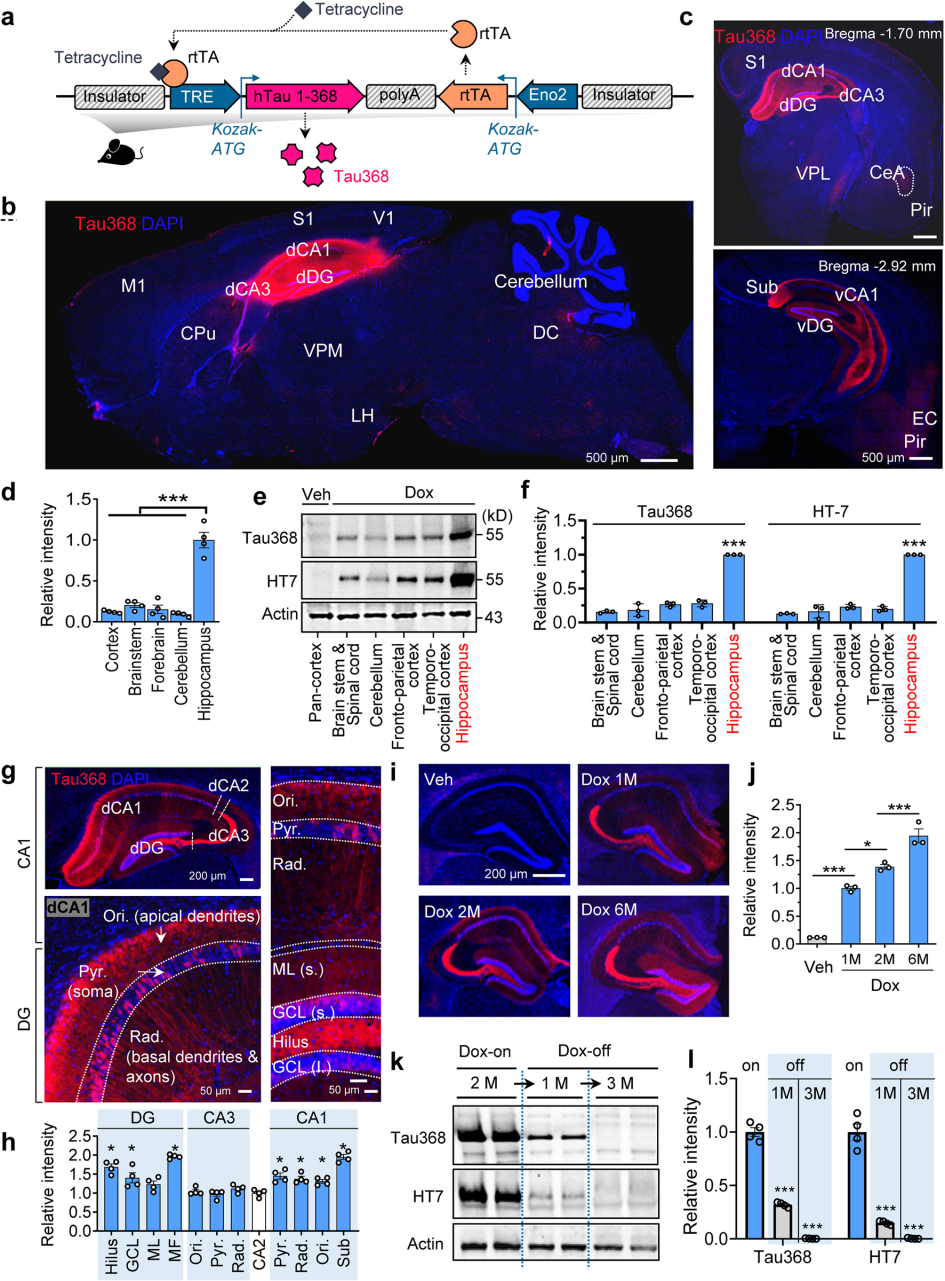
Predominant hTau expression in the hippocampus of dox-administered hTau368 mice. **a** Strategy to express hTau1-368 fragment (hTau368) in transgenic mice. The hTau368 expression was controlled by a tet-on system in combination with the neuronal specific Eno2 promoter. In the presence of doxycycline (dox-on), reverse tetracycline transactivator (rtTA) binds to the tetracycline-responsive element (TRE) to initiate hTau368 expression. In the absence of doxycycline (dox-off), hTau368 expression stops, as rtTA cannot bind to TRE. **b**–**d** Relatively prominent immunofluorescent intensity of hTau368 in the hippocampus following 2-month dox treatment. Representative sagittal (**b**) and coronal (**c**) images are shown. One-way ANOVA followed by Tukey's multiple comparisons tests, *** P < 0.001*, n* = 4 sections from 4 mice. Data were normalized to the mean value in the hippocampus. **e, f** Western blotting showed relatively prominent human tau expression in the hippocampus of dox-treated hemizygous hTau368 mice. One-way ANOVA followed by Tukey’s multiple comparisons tests, *** P < 0.001, compared with the hippocampus; *n* = 3 mice in each group. **g**, **h** hTau368 distribution in both neuronal soma and neurites in multiple hippocampal subregions. One-way ANOVA followed by Tukey's multiple comparisons tests, *P < 0.05, compared with CA2;* n* = 4 sections from 4 mice. Data were normalized to the mean value in CA2. **i**, **j** hTau368 mice (hemizygous) showed time-dependent increase of hTau expression in the hippocampus following dox administration. One-way ANOVA followed by Tukey's multiple comparisons tests, *P < 0.05, ***P < 0.001;* n* = 3 mice in each group, sacrificed at 15–16 months old. Data were normalized to the mean value of the 1-month Dox-treatment group. **k**, **l** The hTau level in the hippocampus of hTau368 mice (hemizygous) gradually decreased during 1–3 months after dox retraction (dox-off). *n* = 4 mice in each group

To design a chimeric transgenic vector, the human *MAPT* gene encoding the hTau368 fragment was placed downstream the tetracycline-responsive element (TRE) promoter, which joined to assemble a tet-on system with a second module expressing the reverse tetracycline-controlled transactivator (rtTA) [31, 32] under the control of the neuron-specific Eno2 (also known as NSE) promoter [33, 34]. These two parts were reversely linked through poly-adenine, and chicken beta-globin insulator was added at both 3′ and 5′ ends to prevent position effects (Fig. 1a). The transgenic vector was constructed and subsequently confirmed by PCR using flanking and insert-specific primers and with restriction analyses. Vectors were transferred into fertilized eggs from C57BL/6 J mice through microinjections, and then engrafted onto the uterus wall of pregnant mice. Successful vector transfection was identified through genotyping in a F0 offspring. Whole-genome sequencing showed that the transgenic vector was randomly inserted into the intron between exons 9 and 10 of gene *Mindy 3* on Chromosome 2. This transgenic mouse was subsequently used for the initial breeding (Additional file 1: Fig. S1).

We next examined the distribution of hTau368 in the brain following dox administration in drinking water (2 mg/l) for 2 months, starting at 2 months of age. Following the 2-month dox treatment, we detected prominent hTau expression in the hippocampus of hemizygous hTau368 mice (Fig. 1b–h), the best-recognized area responsible for cognitive impairments in AD. By contrast, only weak tau signal was detected in other areas of the central nerves system, such as cerebral cortex, forebrain, cerebellum, brainstem and spinal cord (Fig. 1b–h and Additional file 1: Fig. S2). Remarkable hTau aggregation from neurites to soma was observed in hippocampal subregions, including DG, CA3, CA2, CA1 and subiculum (Fig. 1g, h). Also, we found mild hTau368 expression in the entorhinal-piriform cortex (EC and Pir) and amygdala (Fig. 1c and Additional file 1: Fig. S2). Hippocampus, entorhinal cortex and amygdala are vulnerable regions to AD, while cerebellum, brainstem and spinal cord are irrelevant brain areas [35]. These results suggest that in this model, hTau368 is expressed in brain regions susceptible to AD.

Then we focused on hippocampus due to the highest hTau expression level in this region. We found that hTau was accumulated in a time-dependent manner, and dox treatment for one month was enough to induce overt hTau expression in hemizygous hTau368 mice (Fig. 1i, j). Unexpectedly, the hTau expression probed by Tau368 or HT7 antibody (which specifically reacts with hTau proteins) progressively diminished and almost disappeared at 3 months after dox retraction (Fig. 1k, l). We also observed that accumulation of the phosphorylated tau at AT8 (pThr202 & pThr205) epitope was remarkably relieved following dox-off for 3 months, especially in the apical dendrites of CA1 pyramidal neurons and DG (Additional file 1: Fig. S3). These results together indicate that the hTau368 transgenic line is an easy-to-use, inducible and reversible model for studying tau pathologies in AD and related tauopathies, especially when we are focusing on the hippocampus.

### Phosphorylated tau accumulation in the hippocampus of dox-treated hTau368 mice

**Fig. 2:**
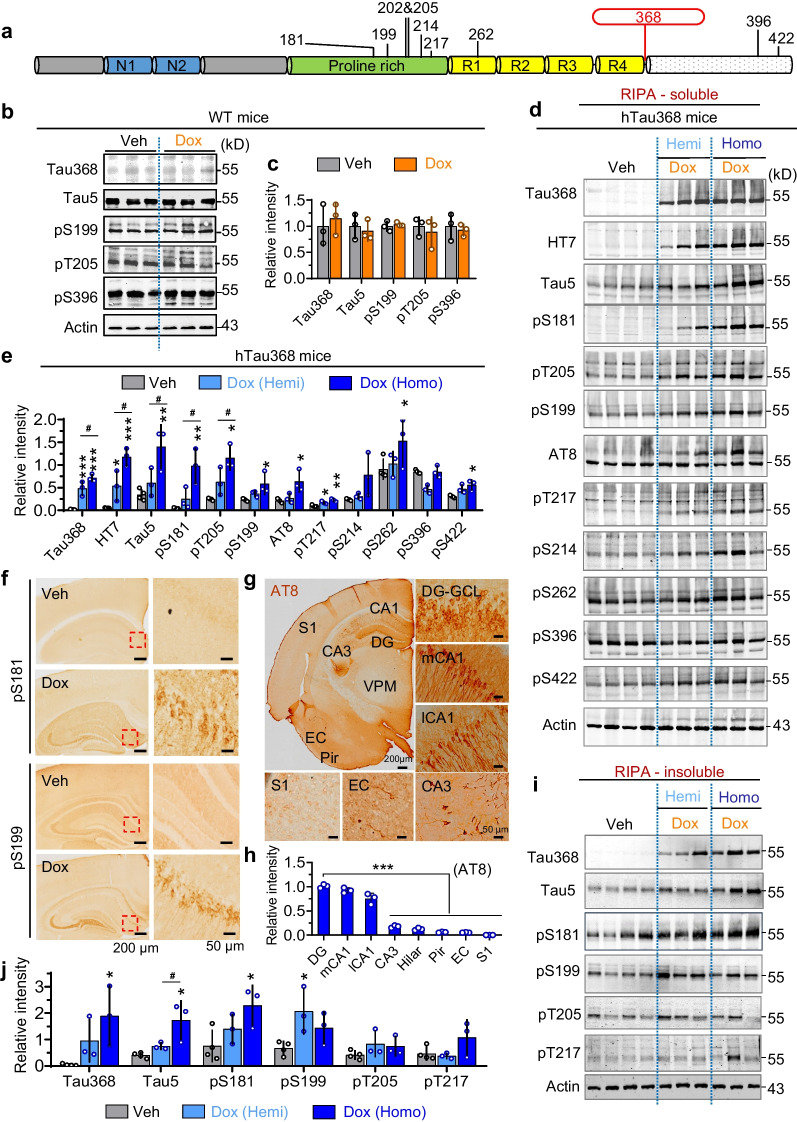
Increase of phosphorylated tau in the hippocampus of dox-administered hTau368 mice.** a** Diagram of human tau protein structure and phosphorylation epitopes measured in this study. **b**, **c** Dox treatment for 2 months showed no influence on tau expression and phosphorylation in wild-type mice. Unpaired Student’s *t*-test, *P* > 0.05, *n* = 3 mice in each group. **d**, **e** Dox-treated hTau368 mice had higher levels of phosphorylated tau in the RIPA-soluble lysate of hippocampus. Homozygotes showed much more prominent pTau increase than hemizygotes. One-way ANOVA followed by Tukey’s multiple comparisons tests, *P < 0.05, **P < 0.01, ***P < 0.001*,* compared with the Veh group (*n* = 4 mice); ^*#*^*P* < 0.05, Dox-Homo (*n* = 3 mice) compared with the Dox-Hemi group (*n* = 3 mice). **f–h** pTau aggregation in the hippocampus of Dox-treated hTau368 mice, detected by immunostaining for pS181, pS199 and AT8 tau. One-way ANOVA followed by Tukey's multiple comparisons tests, ***P < 0.001*, n* = 3 mice in each group. **i**, **j** Dox-treated homozygous hTau368 mice had high levels of pTau in the RIPA-insoluble lysate of hippocampus. One-way ANOVA followed by Tukey's multiple comparisons tests, *P < 0.05, compared with the Veh group, *n* = 3–4 mice in each group

Hyperphosphorylation of tau is the major driver of NFT formation [4]. Over 30 phosphorylation sites have been reported to correlate with tau pathology in AD [36, 37], some of which are regarded as diagnostic markers for AD, such as pTau181, pTau205, and pTau217 [38]. We subsequently examined the level of tau phosphorylation at multiple AD-related epitopes in dox-treated hTau368 mice (Fig. 2a). The WT mice (C57BL/6 J) treated with dox for 2 months did not show changes in the levels of total and phosphorylated tau proteins (Fig. 2b, c). Western blotting detected prominent increases of tau proteins in the RIPA-soluble fraction of hippocampus of dox-treated hTau368 mice, including the truncated tau detected by Tau368 antibody, total tau by Tau5 and HT7, and phosphorylated tau by pSer181, pSer199, pThr205, AT8, pThr217 and pSer422 antibodies. The homozygous hTau368 mice had relatively higher tau levels than their hemizygous siblings at several phosphorylated sites (pSer181, pSer205, and pSer396) (Fig. 2d, e). On the other hand, tau accumulation and hyperphosphorylation was partly (Tau368, Tau5, pSer181 and pSer199) replicated in the hippocampal RIPA-insoluble fraction, but not as prominent as that in the soluble fraction (Fig. 2i, j). In the cortex of hTau368 mice, the dox-induced hTau expression and phosphorylation increased at only some of the epitopes, including Tau368, Tau5, pSer199 and AT8 in the RIPA-soluble fraction (Additional file 1: Fig. S4a), and only Tau368 in the RIPA-insoluble fraction (Additional file 1: Fig. S4b).

Consistently, we also observed evident immunostaining of phosphorylated tau in the hippocampus of dox-treated hTau368 mice using pSer199, pSer181and AT8 antibodies, respectively (Fig. 2f–h, Additional file 1: Fig. S3). The dox-treated hTau368 mice also showed enhanced Gallyas silver staining in DG granular cells, although the intensity was much slighter than that detected in the brain slice of an AD patient (Additional file 1: Fig. S5). Moreover, though pTau mainly accumulated in hippocampal CA1 and DG neurons, EC and Pir also showed slightly positive signals (Fig. 2g, h). Like other tau-related transgenic mice [10, 39, 40], we did not observe significant amyloid deposition in the hTau368 mice after dox treatment for 2 months starting at age 2 months (Additional file 1: Fig. S6).

We consistently detected an increased level of pS422 in the hippocampus and cortex of hTau368 mice (Fig. 2d, e; Additional file 1: Fig. S4), and this site must be from endogenous mouse tau. Consistent with the increased accumulation of the murine phosphorylated tau, we found activation of GSK-3β, an important kinase of tau, as indicated by the decreased ratio of its inactive form (S9 phosphorylated form) to the total GSK-3β, as well as the increased ratio of its active form (Tyr216 phosphorylated form) to the total GSK-3β in the hippocampus of dox-treated hTau368 mice compared with the vehicle controls. In addition, the expression of protein phosphatase-2A (PP2A) and protein phosphatase-1A (PP1A) remained unchanged (Additional file 1: Fig. S7). Indeed, it has been previously reported that the phosphorylation of tau at the Ser422 epitope is indirectly regulated by Tyr216-phosphorylated GSK-3β through Rho and ROCK-signaling [41]. These data suggest that accumulation of the endogenous murine tau may also contribute to the exogenous human truncated Tau368-induced pathologies and cognitive impairments in hTau368 mice.

### Hippocampal neuronal loss and gliosis in dox-treated hTau368 mice

**Fig. 3:**
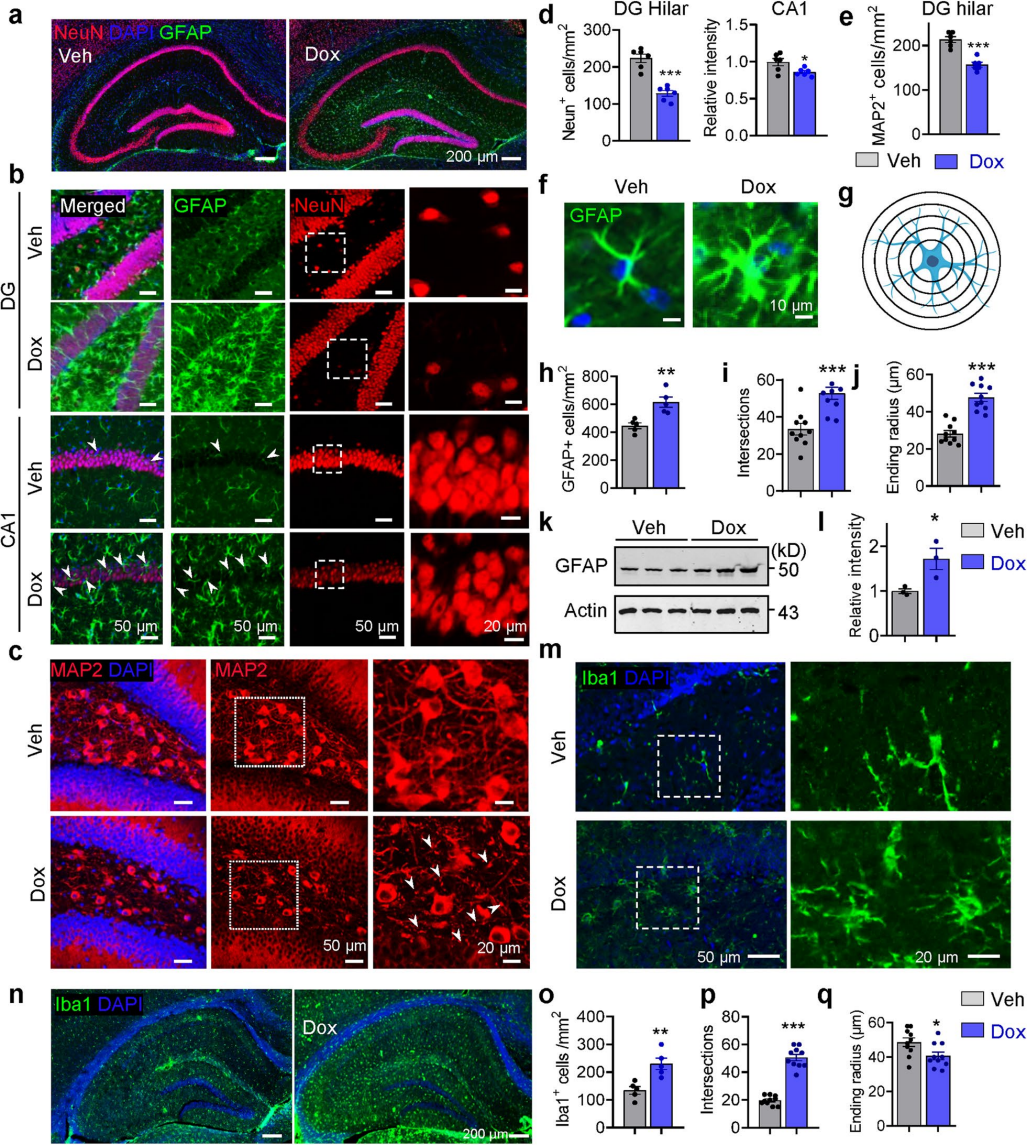
Neuronal loss and glial activation in the hippocampus of Dox-administered hTau368 mice. **a** Representative immunofluorescent images of NeuN- and GFAP-labeled cells in the hippocampus of homozygous hTau368 mice following 2 months of Veh and Dox treatment. **b**–**e** Dox-treated homozygous hTau368 mice had decreased number of NeuN-labeled neurons in DG hilus, relatively lower NeuN immunoreactivity in the CA1 pyramidal layer (**b**, **d**), and decreased number of MAP2-labeled neurons in the DG hilus (**c**, **e**). Unpaired Student’s* t*-test, *P < 0.05, ***P < 0.001; *n* = 6 mice per group. **f** Representative morphology of GFAP-labeled astrocyte. **g** Schematic diagram of Sholl analysis. **h**–**l** Dox-administered homozygous hTau368 mice had increased number and morphological complexity of GFAP-labeled astrocytes in the hippocampus. *P < 0.05, **P < 0.01, ***P < 0.001, unpaired Student’s *t*-test, *n* = 3–5 mice per group. For Sholl analysis, *n* = 10 astrocytes from 5 mice per group. **m**–**q** Dox-administered homozygous hTau368 mice had increased number and morphological complexity of Iba1-labeled microglia in the hippocampus. *P < 0.05, **P < 0.01, ***P < 0.001, unpaired Student’s *t*-test, *n* = 3–5 mice per group. For Sholl analysis, *n* = 10 cells from 5 mice per group

Previous studies have revealed that tau pathology leads to neuronal loss and gliosis in AD [1, 27, 42]. In line with previous findings that hTau368 fragment accumulation is toxic to neurons [25, 29], the dox-treated hTau368 mice showed significant reductions of NeuN-labeled neurons in both DG hilar and CA1 compared with vehicle-treated hTau368 mice (Fig. 3a, b and d). Consistently, significant loss of MAP2-immunoreactive neurons and increased dystrophic neurites were detected in the DG hilar of dox-treated hTau368 mice (Fig. 3c, e).

Gliosis and neuroinflammation play important roles in AD pathology [27]. Compared with the vehicle-treated controls, dox treatment increased the numbers of GFAP-labeled astrocytes (Fig. 3f, h) and levels of GFAP protein to 1.5–2.0 folds in hippocampus (Fig. 3k, l). The astrocytes showed enlarged size and increased process complexity as analyzed by immunostaining and two-dimensional Sholl analysis, respectively (Fig. 3f, g, i and j). The dox-induced expression of hTau368 also caused activation of microglia in the hippocampus, as shown by Iba1-labeled shorter ending radius but more branches related with disease-associated microglia [43] (Fig. 3m-o). Activation of astrocyte and microglia was also detected in EC and Pir following dox-induced hTau368 expression (Additional file 1: Fig. S8), while dox treatment for 2 months exerted very limited effect on glia cells in WT mice (Additional file 1: Fig. S9).

### Neurodegeneration in the hippocampus of dox-treated hTau368 mice

**Fig. 4:**
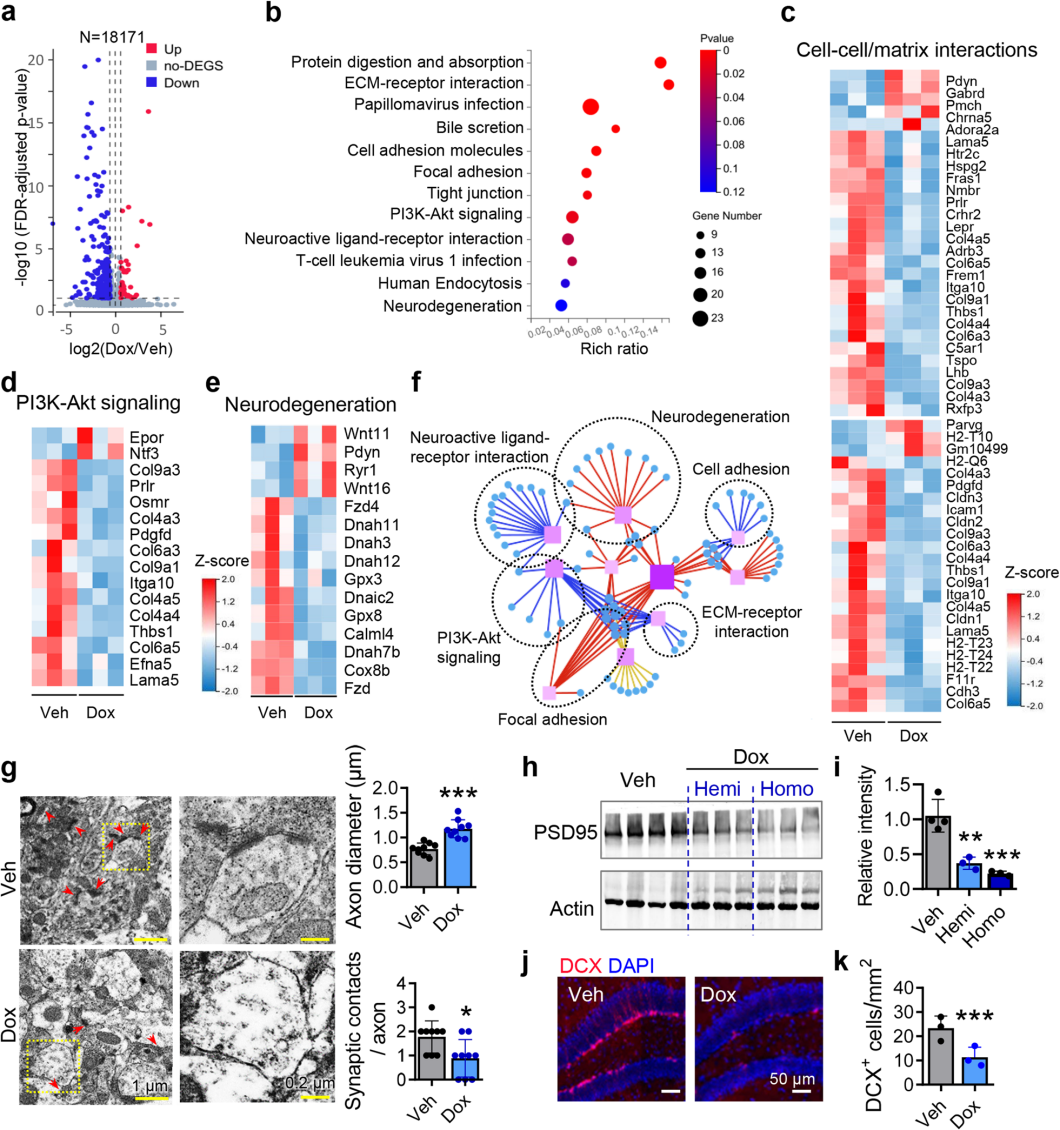
Neurodegeneration in the hippocampus of dox-administered hTau368 mice. **a** Volcano plots illustrating upregulated (red) or downregulated (blue) genes in the hippocampus of homozygous hTau368 mice with 2 months of dox treatment compared with Veh. FDR-adjusted* P* < 0.05 and fold change ≥ 1.5 were considered significant in RNA-seq analysis;* n* = 3 mice in each group. **b**-**e** GESA and KEGG analyses revealed that DEGs were enriched in biological processes involving cell–cell/cell–matrix interactions (ECM-receptor interaction, cell adhesion, tight junction, neuroactive ligand-receptor interaction), PI3K-Akt signaling pathway and neurodegeneration. **f** KEGG pathway relationship network of DEGs. Only the top 10 pathways with the largest number of genes are displayed. Blue dots represent individual genes in each KEGG pathway (purple rectangles) annotated by dashed black circles **g** Dox-treated homozygous hTau368 mice showed axonal swelling and less synaptic contacts. Right panels are zoom-in views of the dashed yellow rectangles in the left panels; red arrows indicate synaptic contacts; *n* = 12 views from 3 mice per group. Unpaired Student’s *t*-test, *P < 0.05, ***P < 0.001. **h**, **i** Dox-treated hemi- and homozygous hTau368 mice had lower levels of PSD95 in the hippocampal tissue. One-way ANOVA followed by Tukey's multiple comparisons tests, **P < 0.01, ***P < 0.001, compared with the Veh group; *n* = 3–4 mice in each group. **j**, **k** Dox-treated homozygous hTau368 mice showed decreased number of DCX-labeled immature neurons in the hippocampus. Unpaired Student’s *t*-test, ***P < 0.001; *n* = 3 sections from 3 mice per group

To characterize molecular changes caused by hTau368 expression in the hippocampus, we performed transcriptomic analysis and identified 467 DEGs, of which 83 were upregulated and 384 were downregulated (Fig. 4a).

Most DEGs were enriched in biological processes of cell–cell and cell–matrix interactions, including extracellular matrix–receptor interaction, cell adhesion, tight junction and neuroactive ligand-receptor interaction (Fig. 4b, c). Besides, many genes involved in the regulation of the PI3K-Akt signaling (a pathway with critical roles in cell survival and AD) like *Epor*, *Ntf3*, and *Col9a3*, were dysregulated [44, 45](Fig. 4d). These imbalanced gene expressions may be attributed to the neurodegeneration resulting from the hTau accumulation (Fig. 4e and f).

Consistently, we observed axonal swellings and less synaptic contacts in dox-treated hTau368 mice by electron microscopy (Fig. 4g), in line with the decreased expression of postsynaptic density protein-95 (PSD-95) (Fig. 4h, i). In parallel with the hTau-induced neurodegeneration, the adult hippocampal neurogenesis was also impaired in dox-treated hTau368 mice, as shown by decreased number of immature neurons labeled by doublecortin (DCX) (Fig. 4j, k). Taken together, these findings further suggest occurrence of neurodegeneration in the hippocampus of dox-treated hTau368 mice.

### Cognitive deficits of dox-treated hTau368 mice

**Fig. 5:**
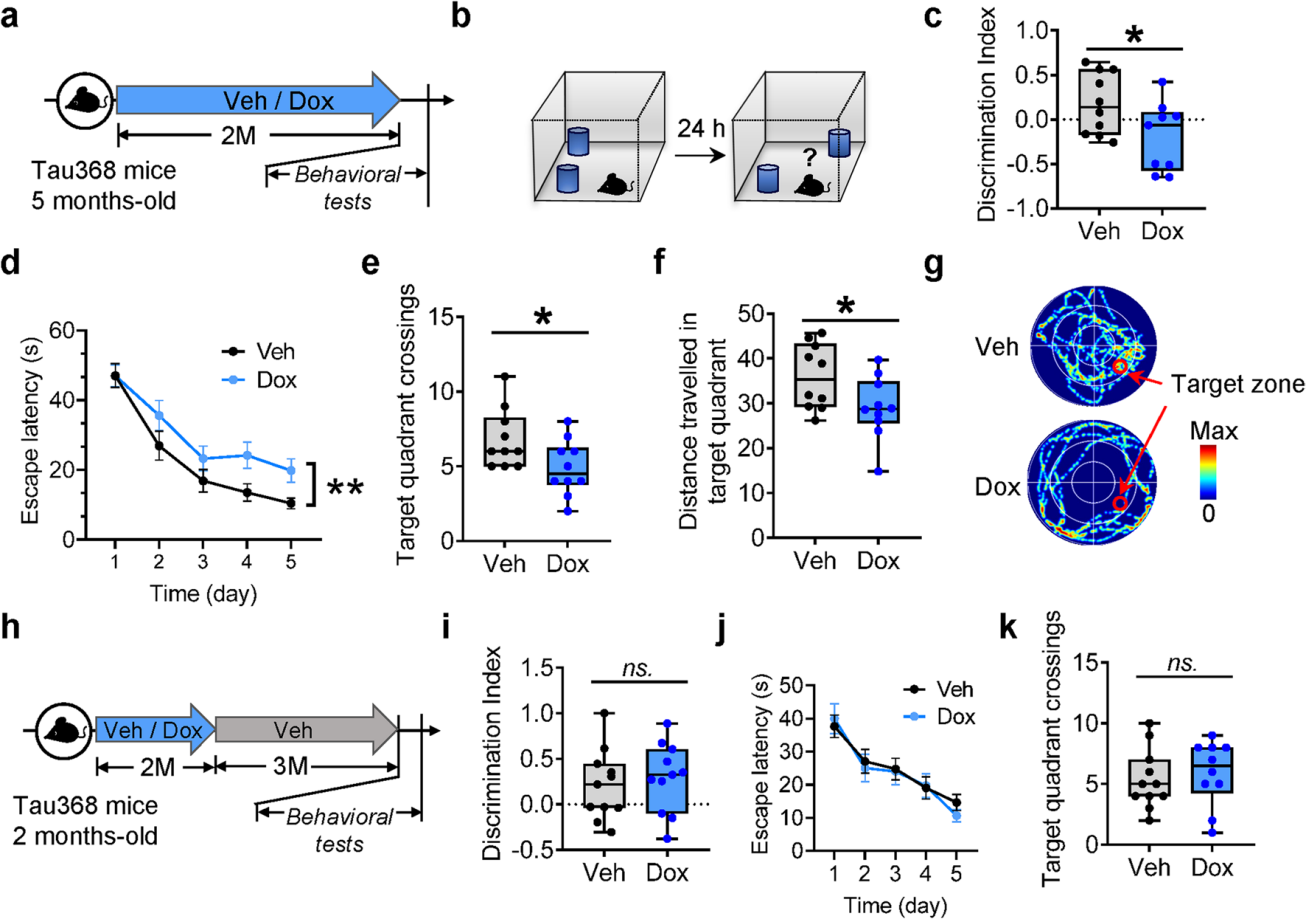
Cognitive deficits of the dox-administered hTau368 mice.** a** Experimental procedure. Behavioral tests were conducted in sex-matched homozygous hTau368 mice following 2-month Veh/Dox administration. **b** Schematic illustration of the procedure of the novel-location recognition test. **c** Dox-treated hTau368 mice showed poorer performance in discriminating the object removed to a new place in the novel-location recognition test. Unpaired Student’s* t*-tests, *P < 0.05; *n* = 9–10 mice in each group **d** hTau368 mice with dox treatment showed lower rate of learning to find the platform in Morris-water maze test. Repeated measures ANOVA followed by Tukey’s *post-hoc* test. **P < 0.01; *n* = 9–10 mice in each group. **e**–**g** hTau368 mice with dox showed decreased time crossing (**e**) and distance travelling (**f**) in the target quadrant when the platform was removed in the Morris water maze. Representative heatmaps (**g**) showing the time and place of mice travelling in the water maze. Unpaired Student’s *t*-tests, *P < 0.05; *n* = 9–10 mice in each group. **h**–**k** hTau368 mice showed no cognitive deficits 3 months after retraction of dox both in the novel-location recognition test (**i**) and the Morris water maze test (**j**, **k**). Unpaired Student’s *t*-test or repeated measures ANOVA followed by Tukey’s *post-hoc* test. *ns.,* nonsignificant*, P* > 0.05; *n* = 10–11 mice in each group

The accumulation of hyperphosphorylated tau is positively correlated with progressive decline of cognitive function in AD [46, 47]. Indeed, we found that in object-place recognition test, the homozygous hTau368 mice with dox treatment showed poorer performance in discriminating objects moved to a new location than the vehicle-treated controls (Fig. 5a–c). In Morris-water maze test, the hTau368 mice exhibited longer latency to find the platform during the acquisition trials (Fig. 5d), as well as reduced times crossing and distances traveling in the target quadrant during the test phase (Fig. 5e–g), indicating impairments in spatial learning and memory. Consistent with the diminishing of tau pathology following dox retraction, the hTau-induced cognitive impairments also recovered at 3 months post dox-off (Fig. 5h–k), accompanied by synaptic remodeling (Additional file 1: Fig. S10) and cessation of the hippocampal neuronal loss (Additional file 1: Fig. S11).

Unexpectedly, we observed that the dox-treated hTau368 mice showed less anxiety-like behaviors, as indicated by the increased entries into the central zone and the open arms in open-field and elevated-plus maze tests, respectively (Additional file 1: Fig. S12). In addition, these mice showed increased locomotor activities than the controls, as evidenced by increased time of exploring and increased distances traveling during the behavioral tests (Additional file 1: Fig. S12). This phenomenon had been seen in other tau-related transgenic mice [24, 48]. The underlying mechanisms remain to be investigated.

Finally, we measured the gender effects on hTau expression and phosphorylation, as well as on cognitive functions in hTau368 mice. Dox induction did not induce significant differences in total tau and pTau levels or in cognitive functions between male and female hTau368 mice (Additional file 1: Fig. S13).

## Discussion

In the present study, we generated a hTau368 transgenic mouse line with inducible and efficient expression of truncated hTau368, which naturally exists in the brain and increases during aging and AD [25, 29, 49]. Dox induced hTau368 expression most predominantly in the hippocampus, the best-recognized area responsible for the spatial learning and memory loss in the early stage of AD, together with AD-like gliosis, neurodegeneration and cognitive deficits. In addition to the hippocampus, minor expression of hTau368 was also detected in other brain regions, including piriform, entorhinal cortex, cortex, cerebellum, brainstem and spinal cord. Coincidentally, prominent tau pathology in the brains of AD patients has been also observed in the entorhinal cortex-hippocampus, from which tau pathology gradually propagates to the limbic system and finally to the whole brain [6, 50]. Therefore, the established hTau368 mouse model in the present study well mimicked the initiation and progression of tau pathology in AD.

To precisely control the starting age and the duration of hTau expression to meet different experimental requirements, we designed the tet-on system to control the neuron-specific Eno2 promoter-driven expression of hTau368. Taking advantage of this system, here we found that dox treatment at the age of 8 weeks, consecutively for 2 months, was sufficient to induce overt tau hyperphosphorylation and accumulation in the hippocampus. On the one hand, starting dox administration in adulthood avoids the potential risks of artificial perturbation of embryonic and infantile development by hTau expression. On the other hand, dox treatment at relatively young ages of adulthood is time- and resource-saving in establishing overt tau pathology, which may otherwise occur at 6–9 months of age in most traditional tau-related mouse models [13, 14, 19]. The highly efficient expression of hTau in the hTau368 mice under dox induction may be due to strong power of TRE in driving gene expression [31].

It should be noted that we used the tet-on system to express the truncated hTau N1-368 in hTau368 mice, instead of intact full-length of WT or mutant hTau as seen in other models. This model has the following advantages. First, the tau N1-368 fragment, generated from AEP cleavage, accumulates in neurons during aging and AD, and shows stronger toxicity to neurons than full-length hTau and other hTau fragments [25, 29]. The truncated tau could interact with full-length tau to gain neurotoxicity [51, 52]. We also previously found that expressing hTau368 in normal C57 mice caused more significant hippocampal neurodegeneration and spatial cognitive impairments than expressing WT full-length hTau [29]. Second, Unlike other types of tauopathy, such as frontotemporal dementia with parkinsonism-17 (FTDP-17) which bears R5L, P301L and R406W mutations in tau [20, 53], to date, no mutations in the tau gene have been identified in AD. Serious motor deficits and reduction of spinal cord motor neurons, associated with neurogenic muscle atrophy and peripheral neuropathy, have been reported in mice with tau mutations at 4–6 months of age, while these symptoms are not observed in patients at early stages of AD [22–24] nor in our hTau368 mouse model. Third, by using the dox-on and off system in this mouse line, precise control of the abnormal tau expression was achieved. Therefore, this mouse model could be used for in-depth exploration of the molecular mechanisms underlying tau accumulation and its neural toxicity, and for tauopathy-related drug development.

However, it should be noted that when measuring tau levels in this hTau368 line, the increases of total and phosphorylated tau appeared to be most prominently at the molecular weight of 55 kD, while previous studies had recognized tau fragments cleaved at N368 at about 15–55 kD, with the most dominant change found at 37 kD [25, 26]. This discrepancy might be partly due to the different antibodies used. In fact, to our knowledge, the accurate molecular weight of tau cleaved at N368 is quite variable and difficult to define. Tau proteins have at least 6 isoforms plus multiple posttranslational modifications, therefore they cover a wide range of apparent molecular mass. In particular, the molecular weight of tau1-368 might vary a lot when AEP or other enzymes cleave 0N4R, 1N4R and 2N4R tau at the same N368 site. Therefore, it is hard to precisely discriminate which blot indicates the tau1-368 fragment simply by Western blotting. Therefore, it would be more objective in future studies to measure all tau bands on the Western blotting membrane, including the 37 and 55 kD blots, to evaluate the tau level when using the hTau368 mouse model.

Several studies have identified “tau filament cores” for its aggregation or toxicity. For instance, a previous study demonstrated that the tau 306–379 region, which contains 10 more amino acids than hTau368, is required for PHF/SF formation in AD [54]. However, it remains unknown whether the additional tau _369_KKIETHKLTF_379_ residues are necessary for the formation of tau filament core. Other studies have revealed a pivotal role of _306_VQIVYK_311_, which is included in the tau 1–368 fragment, in the aggregation of tau [55], and peptides designed against this _306_VQIVYK_311_ fragment are capable of inhibiting tau fibril formation [56, 57]. In postmortem brains of AD patients, tau fragments ending at the N368 site are present in NFTs [58]. These data together strongly support the feasibility of tau 1–368 fragment in mimicking the AD-like tau model.

Unexpectedly, we found here that the tau-associated pathologies and cognitive impairment induced by 2-month dox treatment in hTau368 mice gradually relieved following dox withdrawn for 3 months. This is consistent with a previous report that in a TAU62 transgenic line with dox-dependent 3R tau 151–421 expression, the nerve cell dysfunction and severe paralysis generally recovered when the expression of tau was halted [51]. In another line rTg4510 which uses a tet-off system to control the expression of mutant P301L hTau, tau-associated pathologies like NFTs, neuronal loss, forebrain atrophy and memory impairments were also ameliorated following the cessation of hTau expression [16]. The mechanism for this phenomenon may involve activation of a compensatory system to remove pathological tau, such as activation of the proteasome or lysosomal proteolysis system, and modulation of tau-associated protein kinases and phosphatases which regulate tau phosphorylation and thus indirectly regulate tau degradation. Simultaneously, synaptic remodeling and loss of hippocampal neurons ceased when hTau had been eliminated, which confirmed the toxic role of hTau368. It remains to be determined whether the gradual and autonomic alleviation of tau pathology will still happen when dox-on duration persists for a much longer time, like 6–9 months; the underlying mechanisms warrant further investigations to understand the etiology and unravel the drug targets of AD from the perspective of tauopathy. Additionally, we only presented the results of dox-induction in young animals. As AD is an ageing-associated disorder, the dox-induced tau pathology in older animals needs to be studied. We anticipate that the time to the appearance of similar pathologies and behavioral deficits caused by dox induction should be shorter in older mice.

Moreover, in the brains of the elderly and AD patients, there should be more tau x–368 fragments, which are all cleaved at N368 but at different sites from the C-terminal on the other end. It remains to be defined the content of each fragment in human AD brains, especially the longest 1–368 fragment. We designed the tau368 mice here to express hTau 1–368 since it showed relatively higher cytotoxicity than many other tau fragments [25], while it is also possible for the hTau1-368 fragment to be further cleaved by murine AEP or other endogenous enzymes to generate smaller-size and toxic tau species, like what happens in human AD brains. In addition to the hTau368 mice, other transgenic mouse lines expressing truncated hTau, such as tau159–391 which is also found in AD patients [59], might also be good tools to study tau-associated pathologies in AD.

In previously reported amyloid models, such as PDAPP [60], APPswe/PSEN1dE9 (JAX034832) and 5 × FAD (JAX034848), the age-dependent increase of Aβ pathology is significant while tau pathologies are minor or appear at a relatively old age [36, 61]. As for tau models such as Tau3R0N (hMAPT3R0N, JAX003741), P301L-Tau0N4R (rTg4510, JAX015815 and 016198) and P301S-Tau1N4R (PS19, JAX008169), no significant amyloid deposition is reported [10, 36, 37]. In the present study, we observed that hTau368 mice shared several phenotypes with the amyloid-driven lines, including the increased gliosis, synaptic degeneration and cognitive deficits, but without significant amyloid deposition. These data support that tau pathology could occur independently or downstream of amyloid deposition [62]. Nevertheless, future studies should detect whether induction of hTau368 expression in old animals or its expression for longer times could induce amyloid pathology.

## Conclusion

In summary, here we report a new tet-on transgenic mouse line with inducible and efficient expression of truncated hTau368, which naturally exists in the brains of AD patients and is involved in the progression of neurodegeneration. Following dox treatment, this line shows AD-relevant pathological and behavioral phenotypes. Overall, the hTau368 mouse model is an easy-to-use model for studying the pathogenesis and developing drugs for AD and other tau-related disorders.

### Supplementary Information

**Additional file 1: Fig. S1**. Generation and genomic identification of hTau368 mice.** Fig. S2**. hTau368 had predominantly expression in hippocampus, slightly in other regions.** Fig. S3**. Reversible tau phosphorylation in hTau368 mice following dox-off.** Fig. S4**. Dox treatment increased hTau in the pan-cortex of hTau368 mice.** Fig. S5**. Dox-treated hTau368 mice showed enhanced Gallyas silver staining in DG granular cells, although the staining intensity was much slighter than that detected in the brain slice of AD patients.** Fig. S6**. Dox-treated hTau368 mice did not show amyloid deposition.** Fig. S7**. Dox treatment upregulated GSK-3β activity in the hippocampus of hTau368 mice.** Fig. S8**. Enhanced gliosis in entorhinal-piriform cortex of dox-treated hTau368 mice.** Fig. S9**. Dox treatment showed limited effect on glia activation in wild-type mice.** Fig. S10**. Reduction of tau correlates with increased synapse-associated proteins in hTau368 mice.** Fig. S11**. The loss of hippocampal neurons ceased when dox was retracted for hTau368 mice.** Fig. S12**. Dox-treated hTau368 mice tended to exhibit increased locomotor activities.** Fig. S13**. hTau368 mice showed no gender difference in tauopathy and cognitive behaviors.** Table S1**. Primers used for the identification of hTau368 mice.** Table S2**. Antibodies used in this study.

## Data Availability

All data provided in this paper are available from the leading contact, Prof Jian-Zhi Wang upon reasonable request.

## Abbreviations

EC: Entorhinal cortex
Pir: Piriform cortex
