# Correction: A novel transgenic mouse line with hippocampus-dominant and inducible expression of truncated human tau

**DOI:** 10.1186/s40035-024-00396-y

**Published:** 2024-01-11

**Authors:** Yang Gao, Yuying Wang, Huiyang Lei, Zhendong Xu, Shihong Li, Haitao Yu, Jiazhao Xie, Zhentao Zhang, Gongping Liu, Yao Zhang, Jie Zheng, Jian‑Zhi Wang

**Affiliations:** 1 Department of Pathophysiology, Key Laboratory of Ministry of Education for Neurological Disorders, School of Basic Medicine, Tongji Medical College, Huazhong University of Science and Technology, Wuhan, 430030 China; 2 Department of Neurology, Renmin Hospital of Wuhan University, Wuhan, 430030 China; 3 Key Laboratory of Ministry of Education for Neurological Disorders, Department of Endocrine, Liyuan Hospital, Tongji Medical College, Huazhong University of Science and Technology, Wuhan, 430077 China; 4 Neuroscience Research Institute and Department of Neurobiology, School of Basic Medical Sciences, Peking University , Beijing, China; 5 Key Laboratory for Neuroscience, Ministry of Education/National Health Commission, Peking University, Beijing, 100083 China; 6 Co‑Innovation Center of Neuroregeneration, Nantong University, Nantong, 226000 China

**Correction**: **Translational Neurodegeneration 12:51 (2023)** 10.1186/s40035-023-00379-5

Following publication of the original article [1], the authors reported an error in the Fig. 2:

Figure 2e presented a typing error "HT7" was wrongly written as "HT1". See the Fig. 2 corrected

**Fig. 2:**
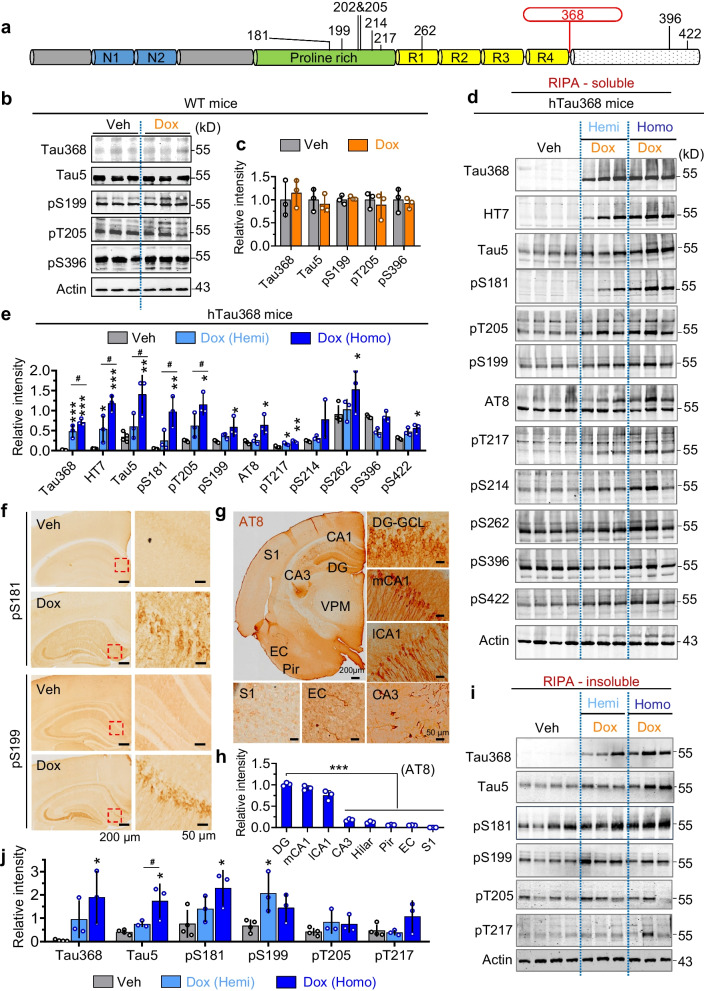
Increase of phosphorylated tau in the hippocampus of dox-administered hTau368 mice. **a** Diagram of human tau protein structure and phosphorylation epitopes measured in this study. **b**, **c** Dox treatment for 2 months showed no infuence on tau expression and phosphorylation in wild-type mice. Unpaired Student’s t-test, P > 0.05, n = 3 mice in each group. **d**, **e** Dox-treated hTau368 mice had higher levels of phosphorylated tau in the RIPA-soluble lysate of hippocampus. Homozygotes showed much more prominent pTau increase than hemizygotes. One-way ANOVA followed by Tukey’s multiple comparisons tests, *P < 0.05, **P < 0.01, ***P < 0.001, compared with the Veh group (n = 4 mice); ^#^P < 0.05, Dox-Homo (n = 3 mice) compared with the Dox-Hemi group (n = 3 mice). **f**–**h** pTau aggregation in the hippocampus of Dox-treated hTau368 mice, detected by immunostaining for pS181, pS199 and AT8 tau. One-way ANOVA followed by Tukey’s multiple comparisons tests, ***P < 0.001, n = 3 mice in each group. **i**, **j** Dox-treated homozygous hTau368 mice had high levels of pTau in the RIPA-insoluble lysate of hippocampus. One-way ANOVA followed by Tukey’s multiple comparisons tests, *P < 0.05, compared with the Veh group, n = 3–4 mice in each group

The original article [1] has been corrected.
