# Supplementary material for: A novel transgenic mouse line with hippocampus-dominant and inducible expression of truncated human tau
Source: Transl Neurodegener. 2023 Nov 10;12:51. doi: 10.1186/s40035-023-00379-5 (PMC10637005; doi:10.1186/s40035-023-00379-5)
Supplement: Supplementary file 1 — Additional file 1: Fig. S1. Generation and genomic identification of hTau368 mice. Fig. S2. hTau368 had predominantly expression in hippocampus, slightly in other regions. Fig. S3. Reversible tau phosphorylation in hTau368 mice following dox-off. Fig. S4. Dox treatment increased hTau in the pan-cortex of hTau368 mice. Fig. S5. Dox-treated hTau368 mice showed enhanced Gallyas silver staining in DG granular cells, although the staining intensity was much slighter than that detected in the brain slice of AD patients. Fig. S6. Dox-treated hTau368 mice did not show amyloid deposition. Fig. S7. Dox treatment upregulated GSK-3β activity in the hippocampus of hTau368 mice. Fig. S8. Enhanced gliosis in entorhinal-piriform cortex of dox-treated hTau368 mice. Fig. S9. Dox treatment showed limited effect on glia activation in wild-type mice. Fig. S10. Reduction of tau correlates with increased synapse-associated proteins in hTau368 mice. Fig. S11. The loss of hippocampal neurons ceased when dox was retracted for hTau368 mice. Fig. S12. Dox-treated hTau368 mice tended to exhibit increased locomotor activities. Fig. S13. hTau368 mice showed no gender difference in tauopathy and cognitive behaviors. Table S1. Primers used for the identification of hTau368 mice. Table S2. Antibodies used in this study. [file 40035_2023_379_MOESM1_ESM.docx]

**Additional file 1**


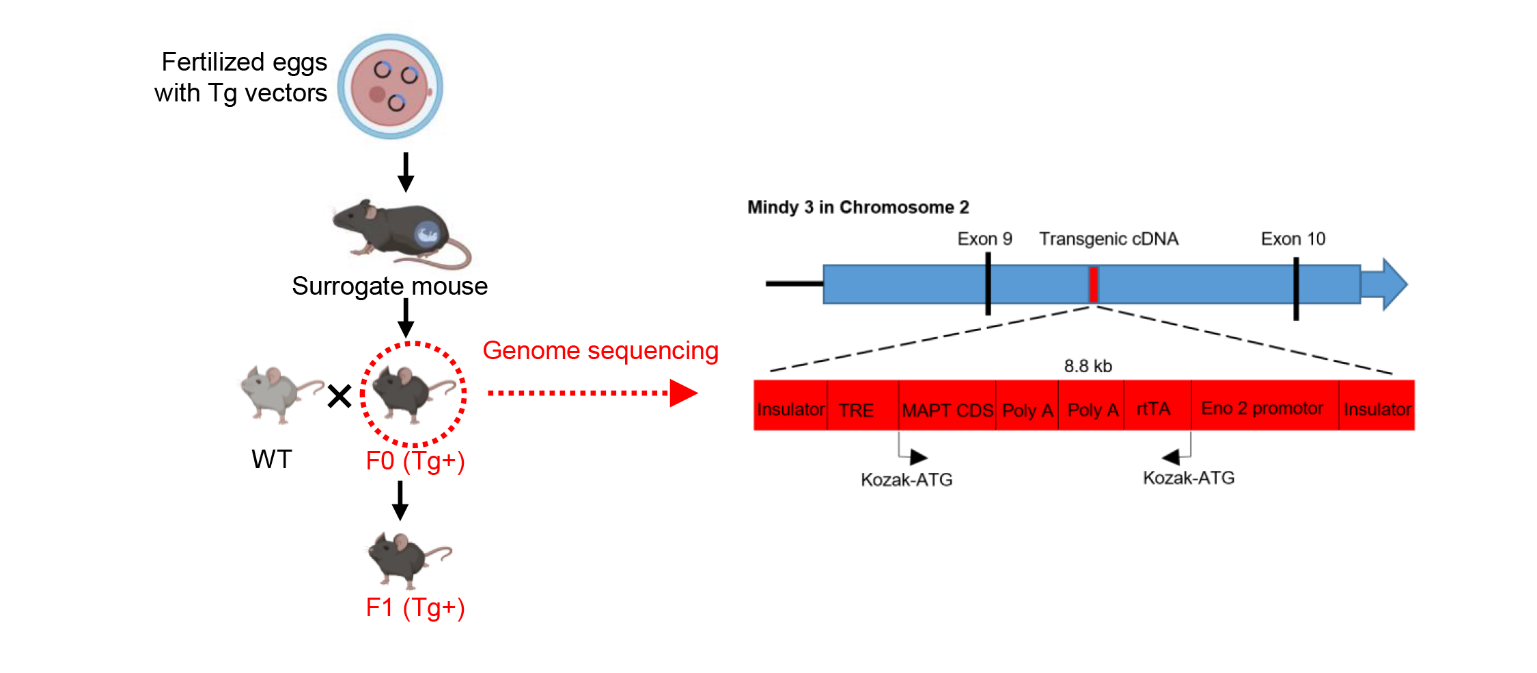


**Fig. S1 Generation and genomic identification of hTau368 mice.** Genomic sequencing revealed that the transgenic vector was inserted into an intron region between the exon 9 and 10 of gene Mindy 3 in Chromosome 2.

**
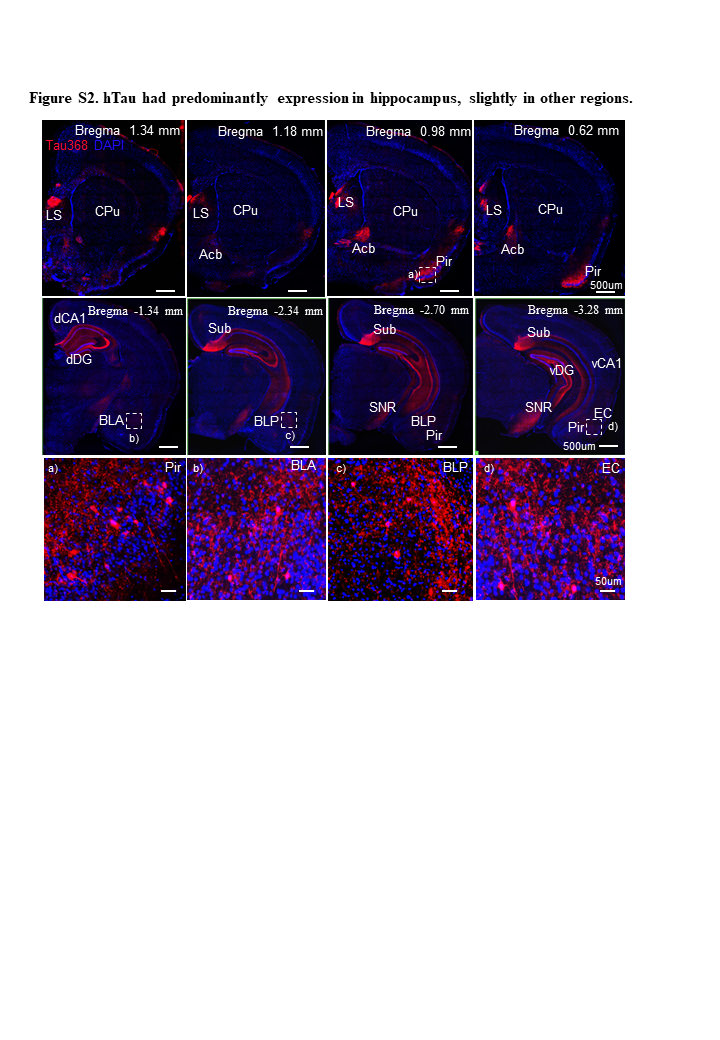
Fig.S2 hTau368 was expressed predominantly in the hippocampus and slightly in other regions.** Coronal views of eight brain slices showing that hTau N1-368 (hTau368) was expressed mainly in hippocampus, while other regions such as entorhinal-piriform cortex (EC and Pir) and amygdala had mild expression. Abbreviation: LS, lateral septal nucleus; CPu, caudate putamen (striatum); Sub, submedius thalamic nucleus; Acb accumbens nucleus; Pir, piriform cortex; SNR, substantia nigra; BLA, basolateral amygdaloid nucleus, anterior part; BLP, basolateral amygdaloid nucleus, posterior part.


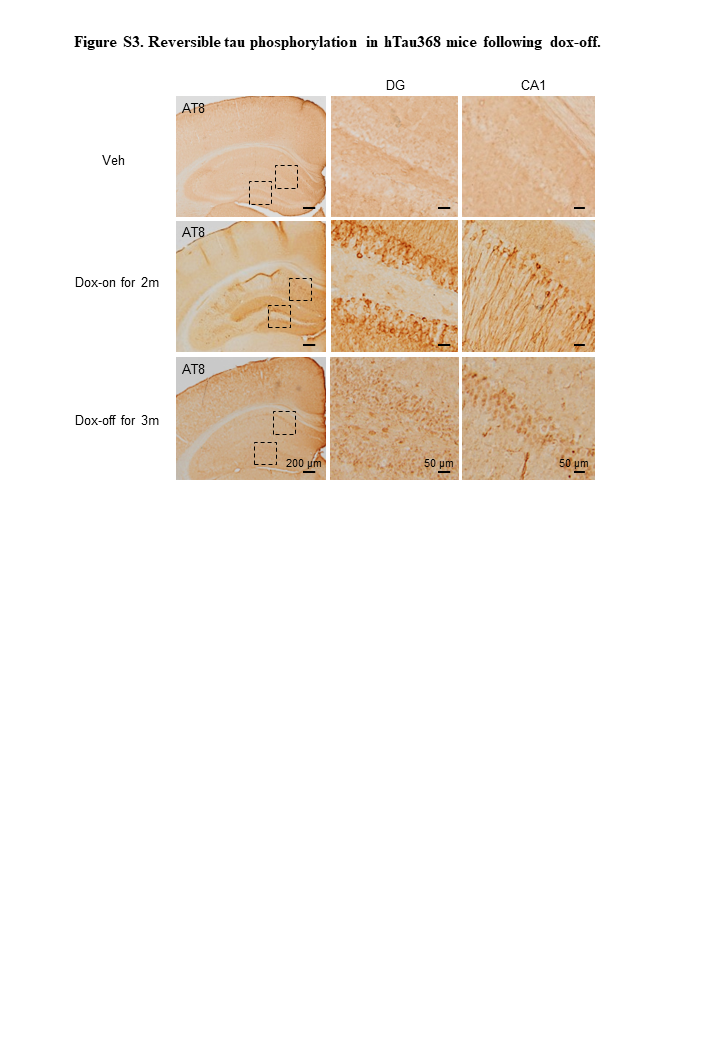


**Figure S3. Reversible tau phosphorylation in hTau368 mice following dox-off.** Representative images of AT8 immumohistochemical staining showed that dox treatment for 2 months induced pTau accumulation in the hippocampus, but pTau was remarkably reduced at 3 months post dox-off.

**
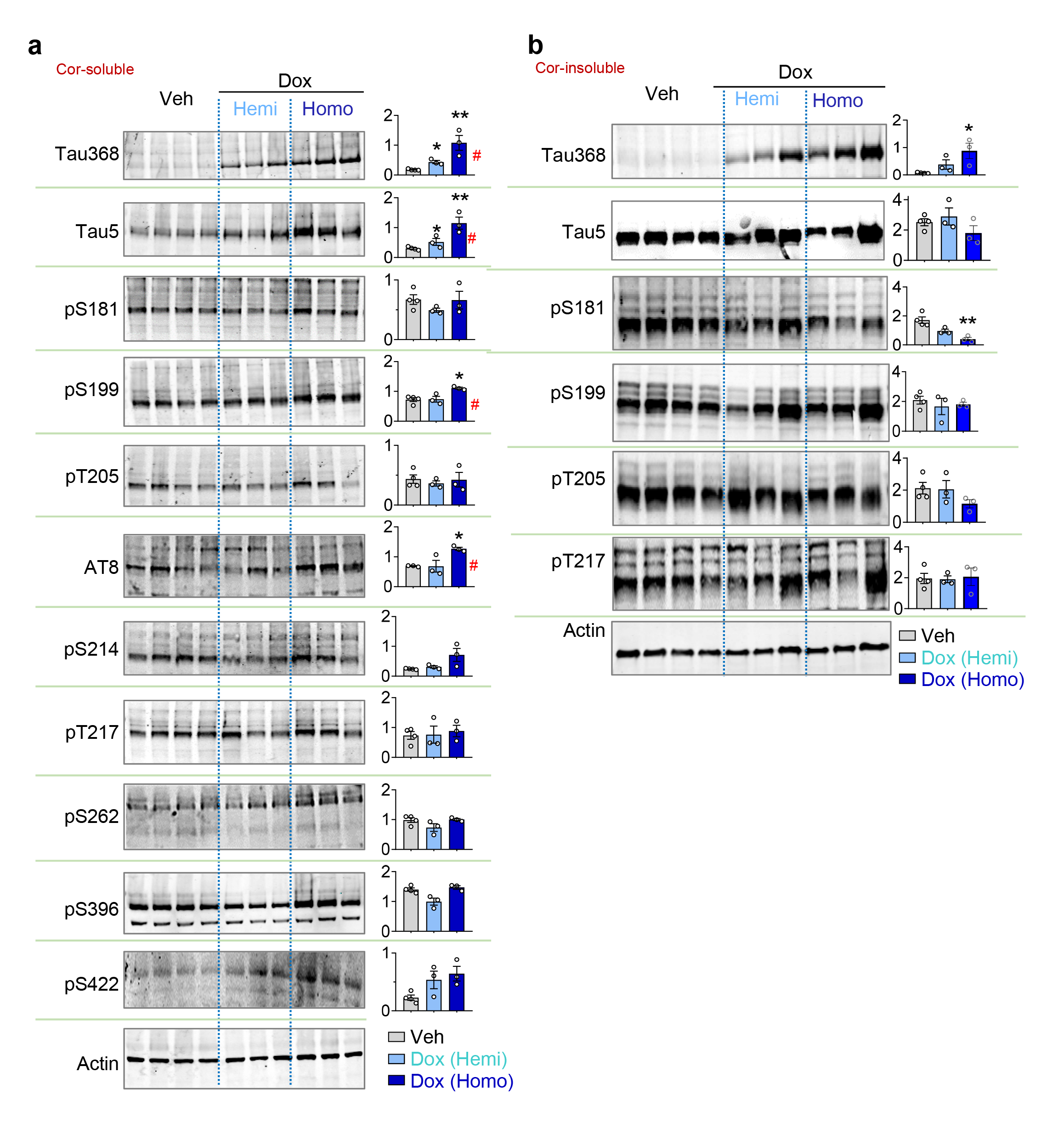
Figure S4. Dox treatment increased hTau in the pan-cortex of hTau368 mice.**

**a** Dox induced hTau368 expression and upregulated tau phosphorylation at some epitopes (pS199, AT8) in the RIPA-soluble lysate of pan-cortex of hTau368 mice. Veh (*n* = 4 mice), Dox-Hemi (*n* = 3 mice), Dox-Homo (*n* = 3 mice). One-way ANOVA followed by Tukey's multiple comparison tests, **P* < 0.05, *** P* < 0.01, **** P* < 0.001*,* compared with the Veh group (*n* = 4 mice); *#p* < 0.05, Dox-Homo (*n* = 3 mice) compared with the Dox-Hemi group (*n* = 3 mice). β-actin was used as the loading control and calculated for intensity normalization.

**b** Dox induced hTau368 expression, but did not increase tau phosphorylation in the RIPA-insoluble component in the pan-cortex of hTau368 mice. Veh (*n* = 4 mice), Dox-Hemi (*n* = 3 mice), Dox-Homo (*n* = 3 mice). One-way ANOVA followed by Tukey's multiple comparison tests, ** P* < 0.05, *** P* < 0.01, compared with the Veh group. β-actin was used as the loading control and calculated for intensity normalization. All values are presented as mean ± SEM.

**
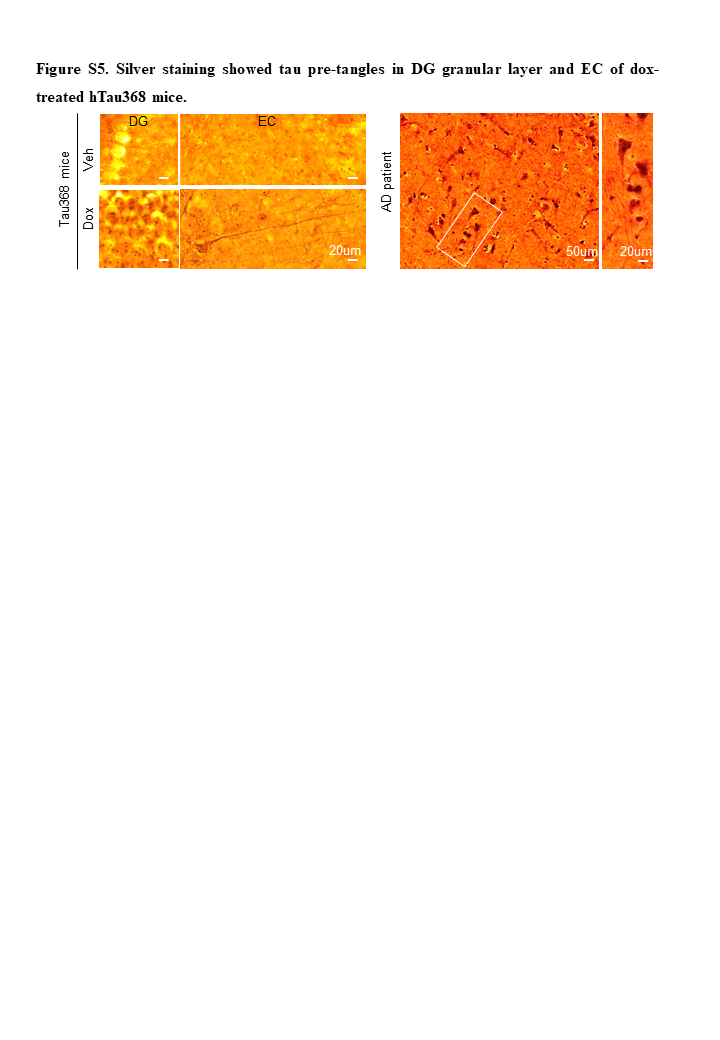
**

**Figure S5.** Dox-treated hTau368 mice showed enhanced Gallyas silver staining in DG granular cells, although the staining intensity was much slighter than that detected in the brain slice of an AD patient.

**
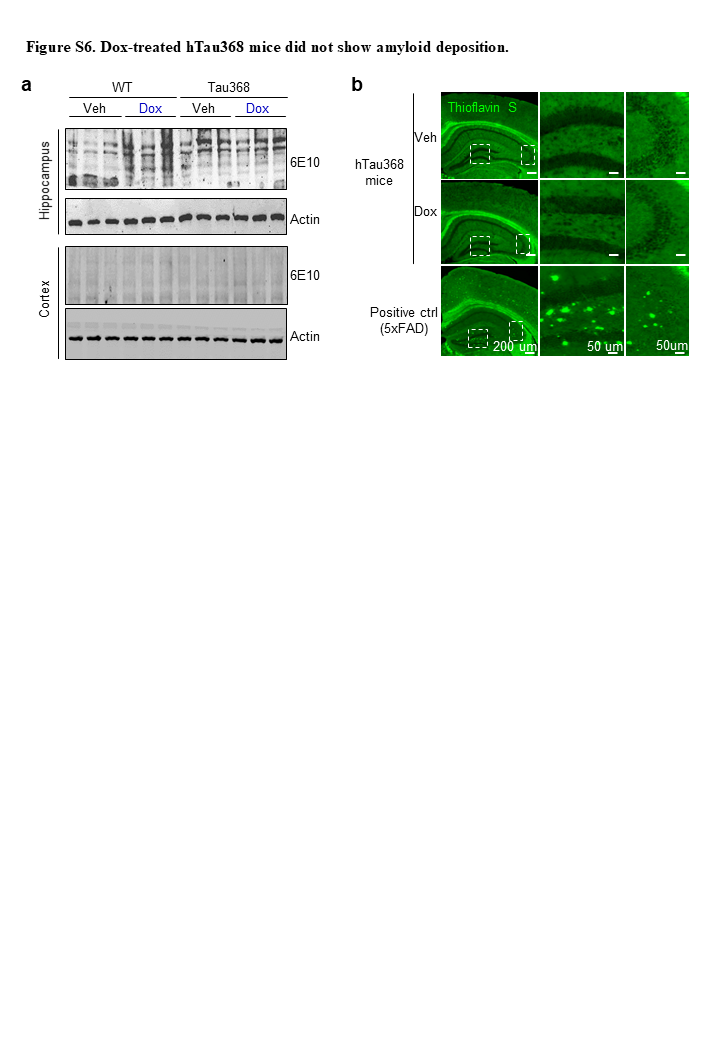
**

**Figure S6. Dox-treated hTau368 mice did not show amyloid deposition.**

**a** The amyloid-beta level was not changed in dox-treated hTau368 mice (for 2 months started at 2 months-old) compared with the Veh controls. *n* = 3 mice in each group.

**b** Thioflavin S staining revealed no amyloid plaques in both dox- and veh-treated hTau368 mice. Slices from 9-month-old 5xFAD mouse were used as a positive control.

**
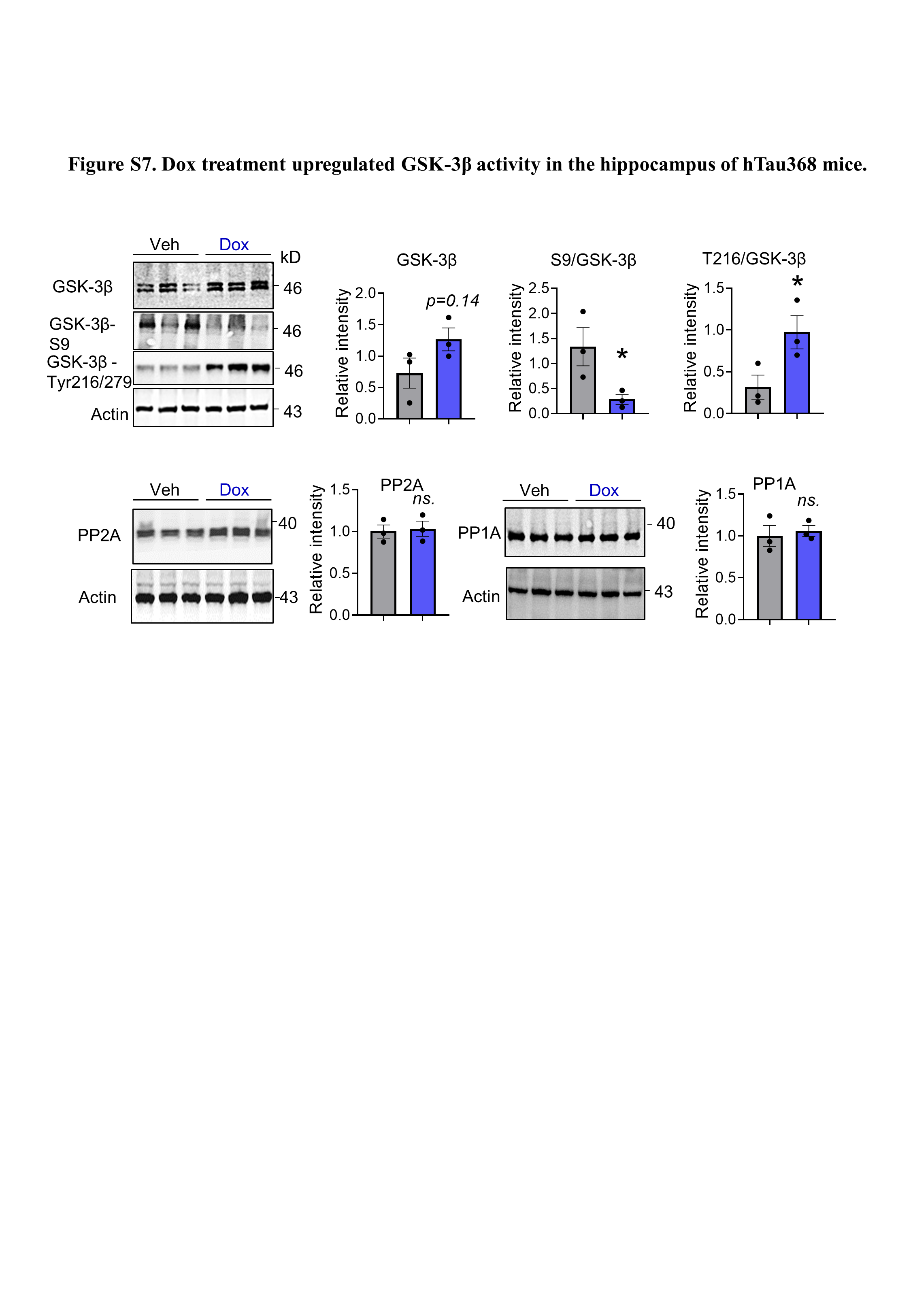
Figure S7. Dox treatment upregulated GSK-3β activity in the hippocampus of hTau368 mice.** The total level of GSK-3β increased, the ratio of GSK-3β-S9 to total GSK-3β decreased, the ratio of GSK-3β-Tyr216/Tyr279 to total GSK-3β increased significantly, while the expression of PP2A and PP1A remained unchanged in the hippocampus of hTau368 mice after 2-month dox treatment. S9-phosphorylation is the inactive form of GSK-3β, while T216/T279 phosphorylation is the active form of GSK-3β. Unpaired Student’s *t*-test, ** P* < 0.05, compared with Veh group, *n* = 3 mice in each group. β-actin was used as the loading control. All values are presented as mean ± SEM.

**
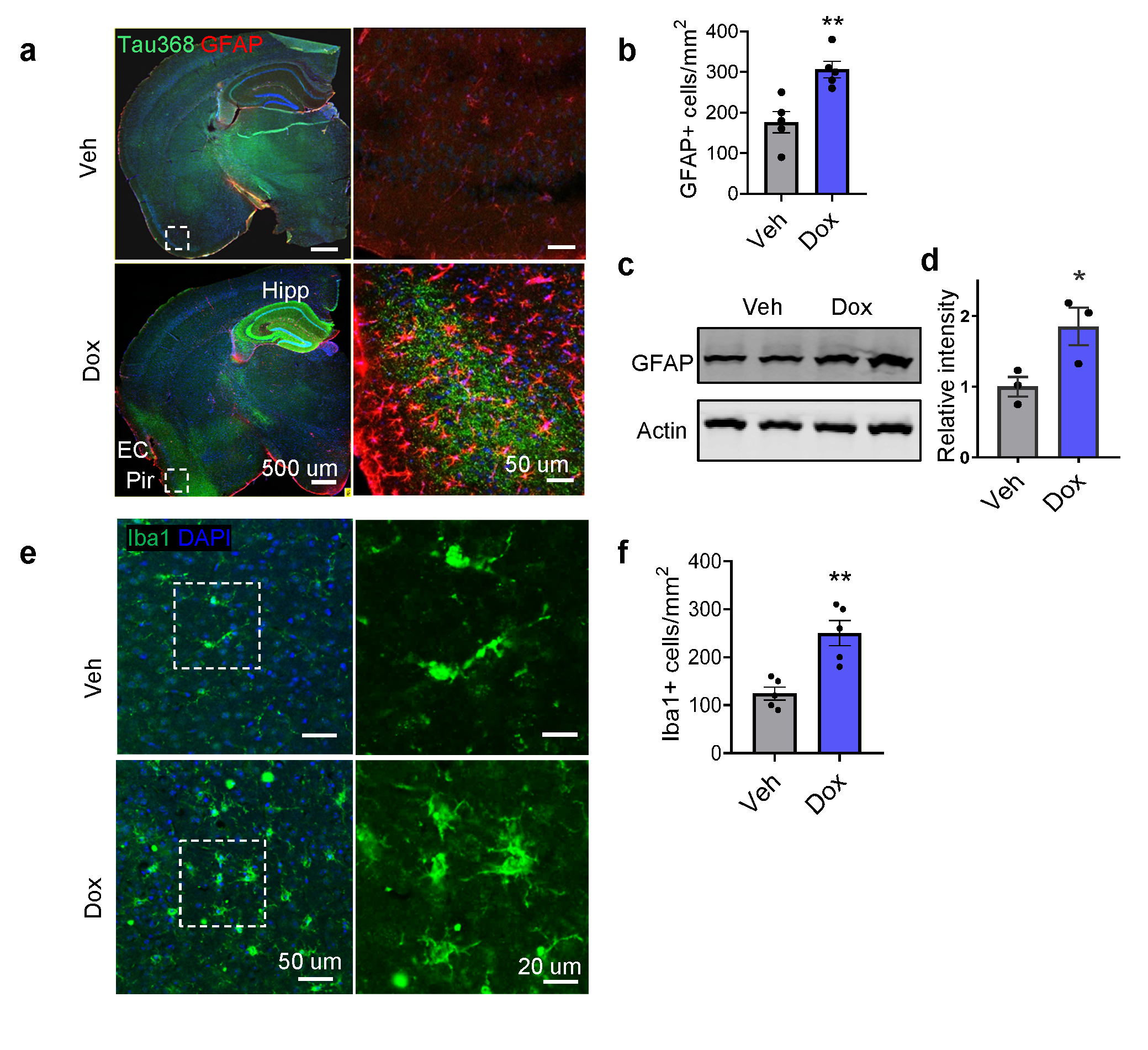
Figure S8. Enhanced gliosis in entorhinal-piriform cortex of dox-treated hTau368 mice.**

**a-d** Dox treatment for 2 months increased the number of GFAP-positive astrocytes (a, b) and GFAP expression level (c, d) in entorhinal-piriform cortex of hTau368 mice. Cells were counted in ×20 power magnification images. Unpaired Student’s t-test, ** P* < 0.05, *n* = 3 mice in each group. β-actin was used as a loading control. Data were normalized to the Veh group.

**e-f** Dox increased the number of Iba1-labeled microglia in the entorhinal-piriform cortex. Cells were counted in 20× power magnification images. Unpaired Student’s *t*-test, ***P*< 0.01, *n* = 5 mice in each group. All values are presented as mean ± SEM.

**
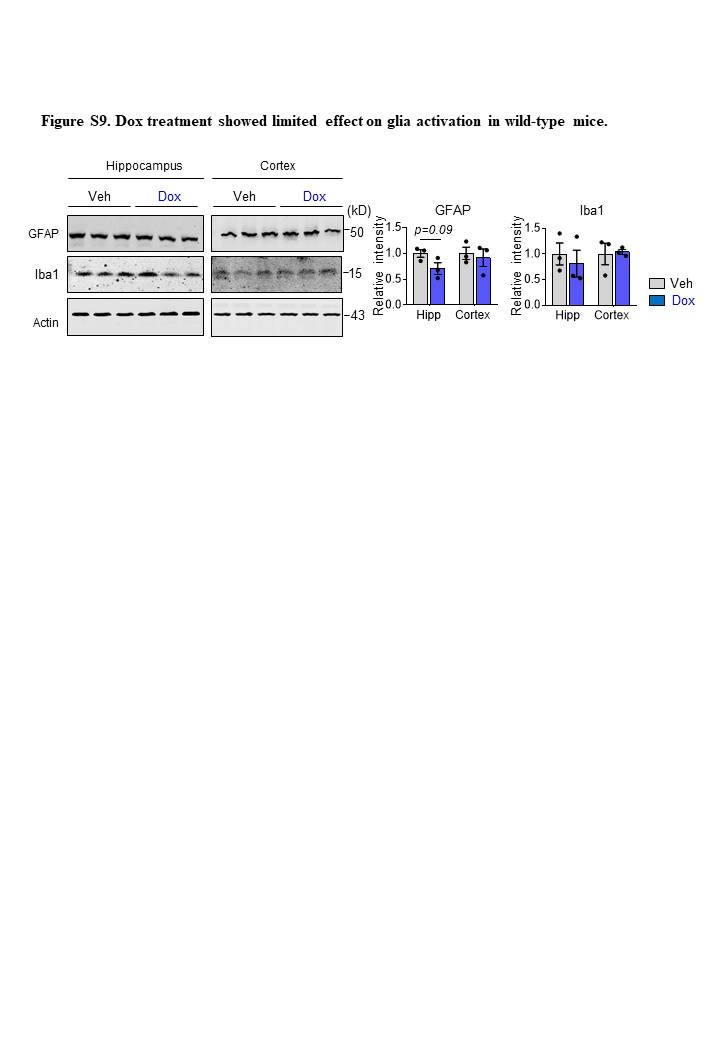
Figure S9. Dox treatment showed limited effect on glial activation in wild-type mice.** Dox treatment did not change the expression of GFAP and Iba1 in the hippocampus and pan-cortex of WT mice. Unpaired Student’s t-test, *P* > 0.05, compared with Veh group, *n* = 3 mice in each group. β-actin was used as a loading control. Data were normalized to the Veh group. All values are presented as mean ± SEM.


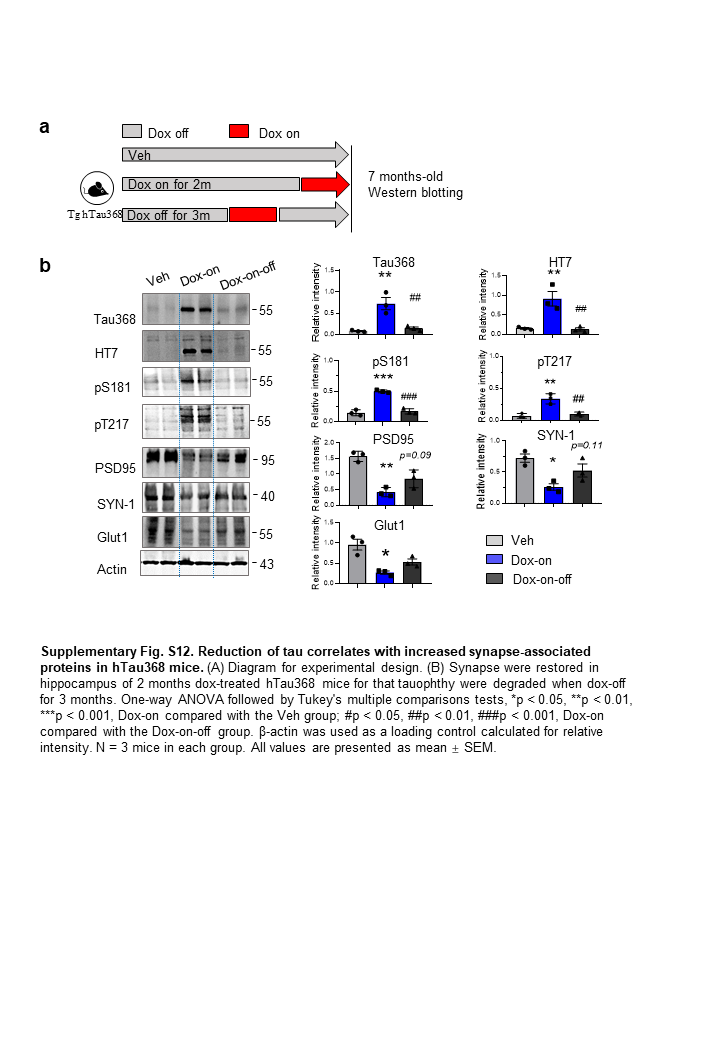


**Figure S10. Reduction of tau correlates with increased synapse-associated proteins in hTau368 mice.**

**a** Diagram for experimental design.

**b** Synapses were restored in the hippocampus of hTau368 mice when tauopathy was degraded after dox-off for 3 months. One-way ANOVA followed by Tukey's multiple comparisons tests, * *P* < 0.05, ** *P* < 0.01, *** *P* < 0.001, Dox-on compared with the Veh group; # *P* < 0.05, ## *P* < 0.01, Dox-on compared with the Dox-on-off group. β-actin was used as a loading control calculated for relative intensity. n = 3 mice in each group. All values are presented as mean ± SEM.


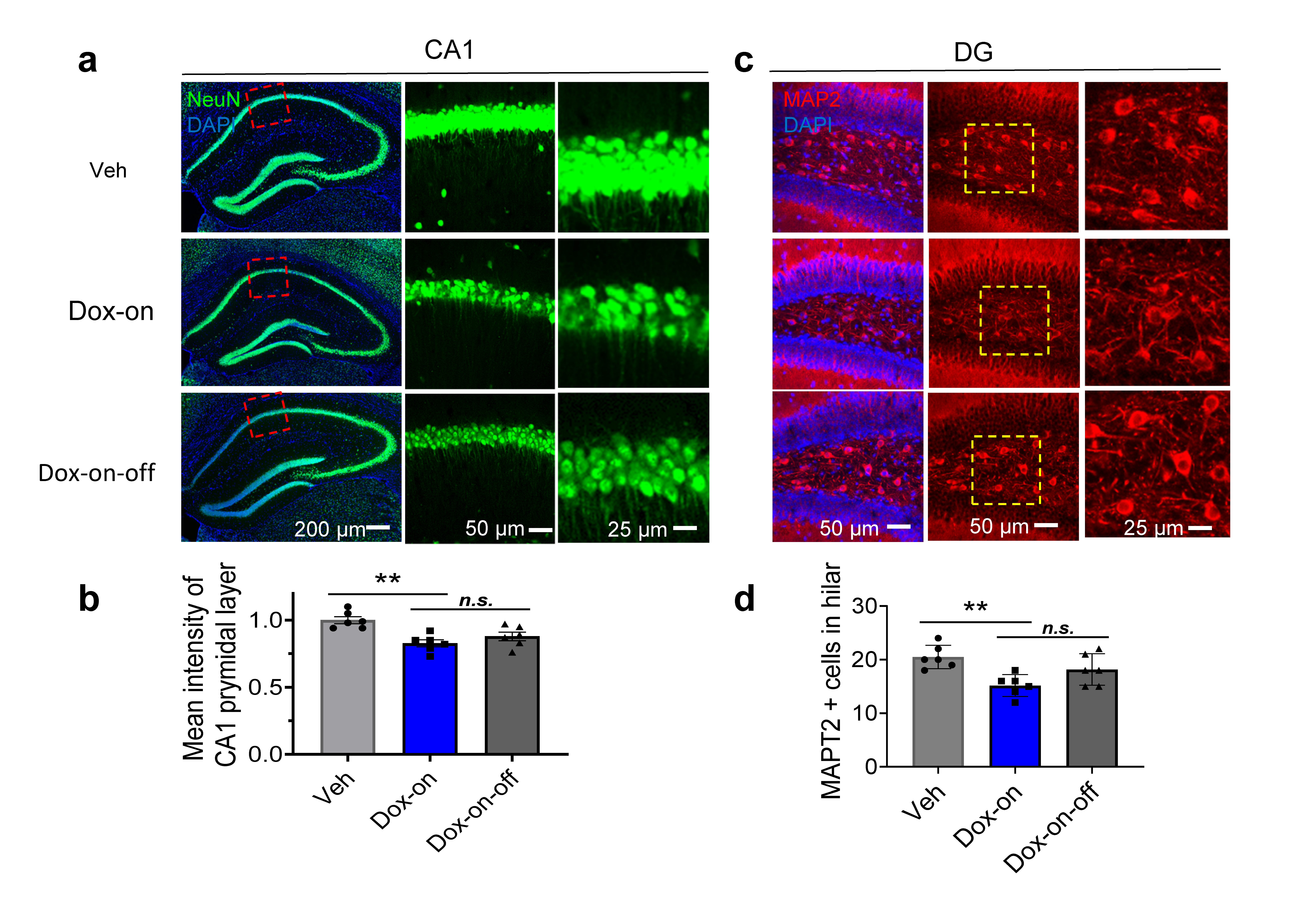


**Figure S11. The loss of hippocampal neurons ceased when dox was retracted in hTau368 mice.** Dox-on-off Tau368 mice showed nonsignificant changes in NeuN staining intensity in the CA1 pyramidal layer (a, b) and in the number of MAP2 immunoreactive cells in the DG hilus (c, d) compared with dox-on mice. One-way ANOVA followed by Tukey's multiple comparisons tests, ** *P* < 0.01, n.s. *P* > 0.05. *n* = 6 mice in each group.

**
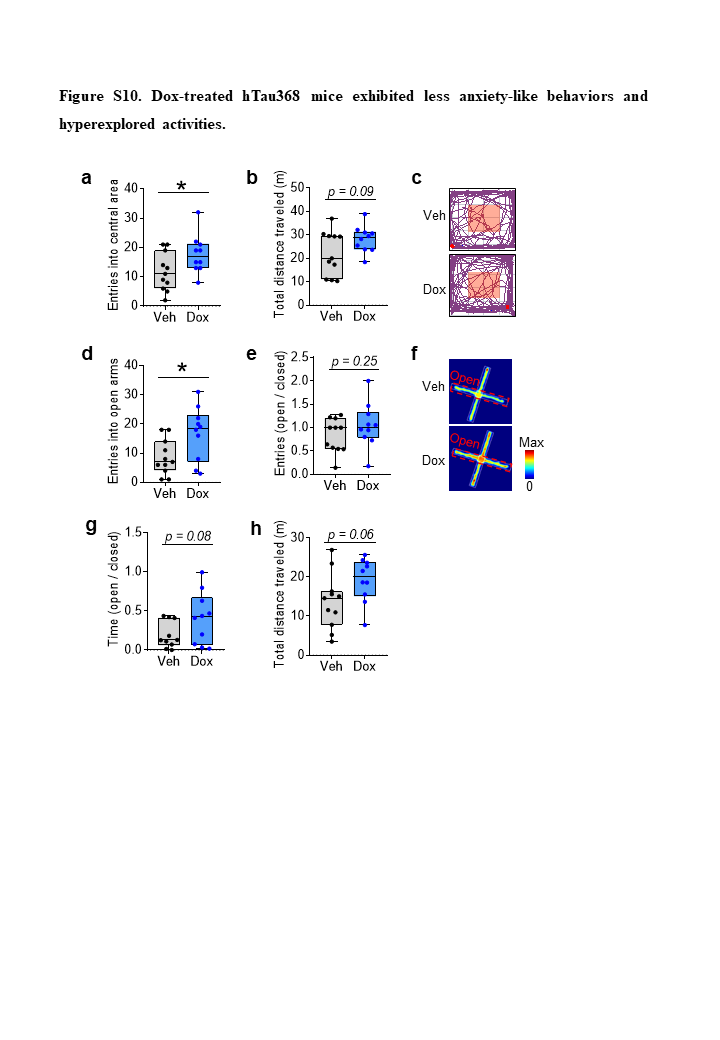
**

**Figure S12. Dox-treated hTau368 mice tended to exhibit increased locomotor activities.**

**a-c** hTau368 mice with dox treatment for 2 months had more entries into the central zone (a), and tended to travel for totally more distance (b) in the open field test. Representative images showed the trajectory of mice travelled in the apparatus (c). Unpaired Student’s t-tests, * p < 0.05. *n* = 11 mice in the Veh group, *n* = 10 mice in the Dox group.

**d-h** hTau368 mice with dox treatment entered more times into the open arms (d), had higher ratios of time spent in the open to that in the closed arms (e) and travelled for totally more distance in the elevated-plus maze test. (**f**) Representative heatmaps showing the time and place of mice travelling in the apparatus. Unpaired Student’s t-tests, * p < 0.05. *n* = 11 mice in the Veh group, *n* = 10 mice in the Dox group. Values are presented as mean (Min, Max).


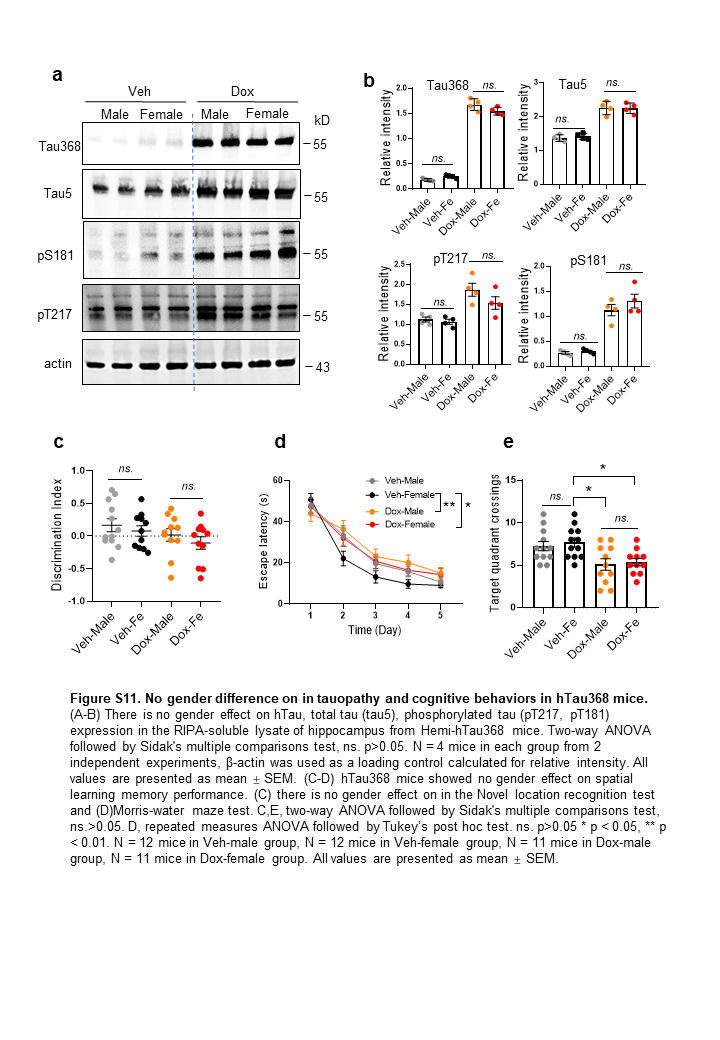


**Figure S13. hTau368 mice showed no gender difference in tauopathy and cognitive behaviors.**

**a-b** There was no gender effect on hTau, total tau (tau5), phosphorylated tau (pT217, pT181) expression in the RIPA-soluble lysate of hippocampus from Hemi-hTau368 mice. Two-way ANOVA followed by Sidak's multiple comparisons test. ns, non-significant; n = 4 mice in each group from 2 independent experiments, β-actin was used as a loading control calculated for relative intensity.

**c-e** hTau368 mice showed no gender effect on spatial learning memory performance. There is no gender effect on in the Novel location recognition test (c) and (d, e) Morris-water maze test. c, e, two-way ANOVA followed by Sidak's multiple comparisons test, ns.>0.05. d, repeated measures ANOVA followed by Tukey’s post hoc test. ns, non-significant. * *P* < 0.05, ** *P* < 0.01. *n* = 12 mice in the Veh-male group, *n* = 12 mice in the Veh-female group, *n* = 11 mice in the Dox-male group, *n* = 11 mice in the Dox-female group. All values are presented as mean ± SEM.

**Table S1 Primers used for the identification of hTau368 mice.**

| ID | Sequence | Product (bp) |
| --- | --- | --- |
| F1 | CTCCAATACGCAGCCCAGTG | 3’-terminal: Tg 742 bp, WT 0 |
| R1 | GTTGCCAGCTTGGTCCTGGA |  |
| F2 | CCAACTTTCCGTACCACTTCC | 5’-terminal: Tg 715 bp, WT 0 |
| F2 | TGGGTGGAGTACGGACCACT |  |
| F3 | CCAACTTTCCGTACCACTTCC | 3’-terminal: Homo/Hemi 151 bp, WT 0 |
| R3 | ACCCTCTTGGTCTTGGTGCATG |  |
| F4 | TGGCCAGCCAAAGTGCTAGT | 5’-terminal: Homo 0, Hemi /WT 453 bp |
| R4 | CAGTCTTTGCCTTGAATGGCAGT |  |

Abbreviations: F, forward. R, reverse. Tg, transgene. WT, wild type. Homo, homozygous. Hemi, hemizygous.

**Table S2. Antibodies used in this study.**

| Antibody | Type | Specificity | Species | Source/reference |
| --- | --- | --- | --- | --- |
| Tau368 | Poly- | N1-368 | R | Gift from Ye^’^s lab [25] |
| Tau368 | Mono- | N1-368 | M | Jointly developed with AtaGenix |
| Tau5 | Mono- | Tau (a.a.210–230) | M | Abcam (ab80579) |
| HT7 | Mono- | Tau (a.a.159–163) | M | ThermoFisher (MN10000) |
| Anti-pS199 | Poly- | p-tau(S199) | R | Invitrogen (44734G) |
| Anti-pT205 | Poly- | p-tau(T205) | R | SAB (11108) |
| AT8 | Mono- | p-tau(S202/T205) | M | ThermoScientific (MN1020) |
| Anti-pT217 | Poly- | p-tau(T217) | R | SAB (11724) |
| Anti-PT181 | Poly- | p-tau(T181) | R | SAB (11107) |
| Anti-pS214 | Poly- | p-tau(S214) | R | Invitrogen (44-742G) |
| Anti-pS262 | Poly- | p-tau(S262) | R | Abcam (ab80579) |
| Anti-pS396 | Poly- | p-tau(S396) | R | Abcam (ab32057) |
| Anti-pS422 | Poly- | p-tau(S422) | R | Abcam (ab79415) |
| PSD-95 | Poly- | PSD-95 | R | SAB (41365) |
| GSK-3β | Poly- | GSK-3β | R | SAB (38353) |
| GSK-3β S9 | Mono- | p-GSK-3β(Ser9) | M | CST (D2Y9Y) |
| GSK-3β Tyr216/279 | Mono- | p-GSK-3β(Tyr216/279) | M | Upstate (32160702) |
| β-Actin | Mono- | β-Actin | M | SAB (21800) |
| Dylight 800 |  | Dylight 800 | R | Immunoway (RS23920) |
| Dylight 800 |  | Dylight 800 | M | Immunoway (RS23910) |
| NeuN | Mono- | NeuN | R | CST (24307) |
| MAP2 | Mono- | MAP2 | R | Millipore(AB5622) |
| GFAP | Mono- | GFAP | M | CST (3670) |
| Iba1 | Mono- | Iba1 | G | SAB (ab5076) |
| Doublecortin | Poly- | Doublecortin | M | SAB (ab18723) |

Antibody dilutions: 1:200 for immunostaining; 1:1000 for Western blotting.

Abbreviations: Mono-, monoclonal: p-, phosphorylated; Poly-, polyclonal: M, mouse; R, rabbit.
